# High genomic connectivity within *Anatoma* at hydrothermal vents along the Central and Southeast Indian Ridge

**DOI:** 10.1038/s41598-025-85507-z

**Published:** 2025-01-15

**Authors:** Katharina Kniesz, Leon Hoffman, Pedro Martínez Arbizu, Terue C. Kihara

**Affiliations:** 1 Senckenberg am Meer, Wilhelmshaven, Germany; 2 Carl von Ossietzky Universität Oldenburg, Oldenburg, Germany; 3 Leibniz-Institut für Ostseeforschung Warnemünde, Rostock, Germany; 4 INES Integrated Environmental Solutions UG, Wilhelmshaven, Germany

**Keywords:** Gastropoda, Connectivity, Hydrothermal vents, Indian Ocean, COI, 2b-RAD, Population genetics, Marine biology

## Abstract

Hydrothermal vents are ecosystems inhabited by a highly specialized fauna. To date, more than 30 gastropod species have been recorded from vent fields along the Central and Southeast Indian Ridge and all of them are assumed to be vent-endemic. During the INDEX project, 701 representatives of the genus *Anatoma* (Mollusca: Vetigastropoda) were sampled from six abyssal hydrothermal vent fields. Traditional morphology and COI barcoding of Hoffman et al. (Eur J Taxon 826:135–162, 2022) were combined with 2b-RAD sequencing to investigate the anatomid community structure and connectivity between the different vent fields. Consequently, 2b-RAD sequencing supported the primary species hypothesis (based on morphology) for 125 individuals of the recently described taxa *A. discapex*, *A. declivis*,* A. laevapex* and *A. paucisculpta*. We assigned 22 additional specimens to species with 2b-RAD sequencing and updated the community analyses that confirmed the pattern of expanding populations. Population structure and F_ST_ values indicated high connectivity along the six sampled vent fields for the three most abundant species. High levels of gene flow are suggested, pointing to high dispersal potential of the target species along the study area. However, low levels of heterozygosity revealed a small gene pool and therefore an increased vulnerability towards environmental change. Our results demonstrate that 2b-RAD sequencing, in combination with other molecular methods, can accurately characterise macrobenthic mollusc communities. Sequencing technology is an essential tool for ongoing monitoring. Furthermore, we highlight that the inferred molecular and ecological patterns provide valuable insights into hydrothermal vent ecosystems, which are crucial for the successful conservation of these ecosystems.

**Supplementary Information:**

The online version contains supplementary material available at 10.1038/s41598-025-85507-z.

## Introduction

A recent agreement among members of the United Nations has resulted in the formulation of the High Seas Treaty to protect biodiversity in international waters. The objective is to ensure the protection of at least 30% of international waters by 2030 ^2^. This new agreement demonstrates that the marine environment and its inhabitants are susceptible to adverse human impacts from commercial fishing, shipping, pollution and climate change. The deep seabed is also becoming increasingly economically interesting to the gas, oil and mineral industries. Moreover, future human activities will affect the deep-sea habitats of manganese nodules and massive sulphides at hydrothermal vent systems^3–5^. It is important to study the present fauna and its unique position in these ecosystems to define mitigation measures for detrimental industrial activities and to facilitate nature conservation.

To ensure the protection of hydrothermal ecosystems under potential human intervention, besides documenting the distribution of the local fauna, their dispersal potential and genetic connectivity must also be considered. Specific environmental conditions facilitate or hinder dispersal at hydrothermal vents, including biotic (larval longevity, feeding mode, physiology and behaviour) and abiotic factors (circulation, water column density, oxygen levels, hydrothermal plume geochemistry)^6^. However, the most widely recognized driver is the duration of the planktonic stage^7^. Although, a longer larval stage may facilitate a higher potential for dispersal^6^, it does not necessarily result in an elevated recruitment or survival rate of species at hydrothermal vents. For instance, prolonged larval residence in currents increases the likelihood of being transported off-axis and therefore missing the species’ intended habitat^8^.

Species connectivity at hydrothermal vent fields has been studied in the Indian Ocean ridge system, along the Central Indian Ridge (CIR), Southwest Indian Ridge (SWIR) and Carlsberg Ridge (CR). Studies on genetic connectivity are only lacking on the Southeast Indian Ridge (SEIR). Previous connectivity studies focusing on gastropods (*Alviniconcha* spp., *Chrysomallon squamiferum*) and decapods (*Rimicaris kairei* and *Austinograea rodriguezensis*) revealed no genetic differentiation along the CIR, suggesting a high dispersion ability of the species examined^9–12^. In contrast, genetic isolation is observed between the CIR and the SWIR populations^11,13,14^, suggesting that transform faults are the main barrier between these populations^13^. Recent studies have analysed populations from the northernmost part of the Indian Ridge system, the CR, and concluded that these populations are significantly different from CIR populations for *Neoplepas marisindica*, *Chrysomallon squamiferum*, *Bathymodiolus septemdierum* and *Hesiolyra heteropoda*^14^. They suggest that the Indian Ocean vents should be treated as three provinces for conservation purposes.

To evaluate the feasibility and impacts of deep-sea massive sulphide mining, the Indian Ocean Exploration (INDEX) project conducts biodiversity inventories and connectivity studies of hydrothermal vent ecosystems along the CIR and SEIR. As part of the INDEX project, Hoffman et al.^1^ identified six species of the genus *Anatoma* (family Anatomidae) from abyssal hydrothermal vent environments by mitochondrial DNA (COI, Cytochrome oxidase subunit I) analysis and described four new species based on morphological characteristics: *Anatoma discapex*, *A. declivis*, *A. laevapex* and *A. paucisculpta*. The remaining two species are evidently distinct yet remain undescribed since they are represented by a single individual each. The species *A. paucisculpta* forms a sister group to the undescribed species *Anatoma* sp. Lau (GenBank accession number: AB365210)^15^ from hydrothermal vents in the Lau Basin, Pacific Ocean^1^. Moreover, some specimens in the study remain unidentified due to the damage of the shells and the failure of barcoding COI, which is a consequence of the fixation and age of the samples.

In the present study, we aim to investigate the genetic connectivity of these anatomid species identified from Hoffman et al.^1^ across six sampled hydrothermal vent fields along the CIR and SEIR using both genetic barcode and genome-wide data. The question is of particular importance with respect to potential future deep-sea mining in the study area. We use 2b-RAD (restriction-site associated DNA with type IIB restriction endonucleases) sequencing to analyse the *Anatoma* species with regard to the former species delimitation and to identify additional specimens. Furthermore, we compare the genomic dataset with the previously published COI data, examine population structure and genetic diversity. In addition, we present the distribution of the genus *Anatoma* along the CIR and SEIR.

## Materials and methods

### Sampling and sample treatment

**Fig. 1:**
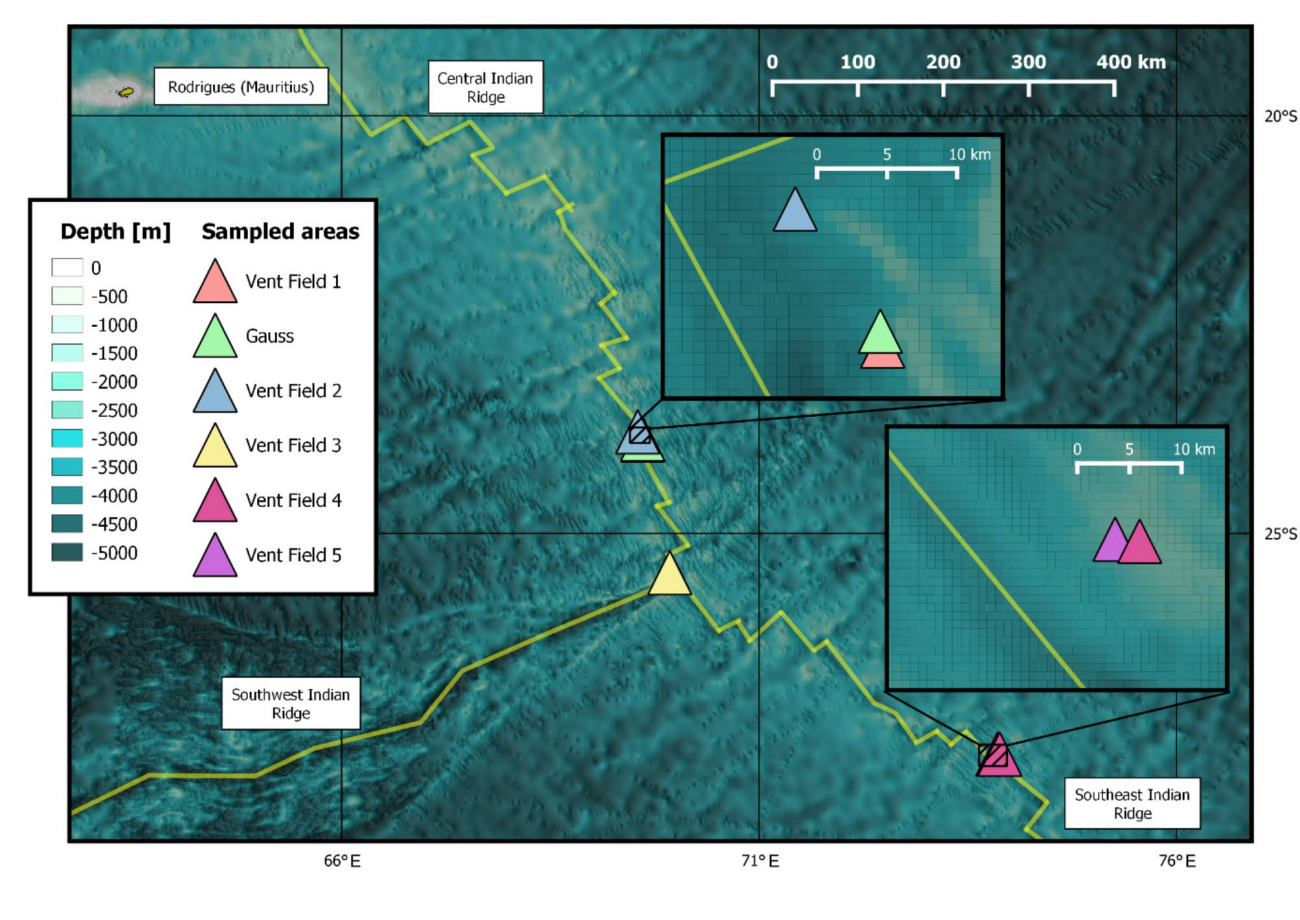
Six sampled vent areas in the Central Indian Ocean. Map is showing the mid ocean ridges: Central, Southwest and Southeast Indian Ridge. Map by QGIS using data from BGR (Bundesanstalt für Geowissenschaften und Rohstoffe), Hannover.

The material used in this study was sampled in the Indian Ocean along the CIR and SEIR in six hydrothermal vent areas (Fig. 1) during three cruises of the INDEX project. Samples were collected using the Canadian ROV ROPOS, mainly by rock picking or suction sampling close to the vent fields.

This study is based on the data published in Hoffman et al.^1^, where a total of 701 anatomids were handpicked from the samples. A subset of 169 specimens was chosen for COI barcoding, resulting in 95 high-quality sequences and six Molecular Operational Taxonomic Units (MOTUs). Subsequently, the species were morphologically studied, confirming the molecular identification, thereby resulting in the description of four new species. The analytical methodology used for COI barcoding is given in Hoffman et al.^1^. A comprehensive specimen list and sampling details are available in the supplementary information of this study (Supplementary Table S1).

The study of anatomids was impeded by two factors: the loss of the shell during DNA extraction and the age and fixation of the specimen material. Morphological identification of some specimens was almost exclusively based on low resolution microscope images, which introduced a considerable degree of uncertainty. In addition, differences in the way specimens were handled during the three cruises resulted in varying success rates for COI barcoding^1^, with 53.2% of the specimens from cruise INDEX2015 remaining without barcode. Specimens from INDEX2015 were left in the sediment for one year after fixation in 96% undenatured ethanol. They stayed at room temperature and may have been warmed by transport under tropical conditions (temperatures can reach 30–60 °C in the transport container^16,17^). In comparison, the samples from INDEX2018 and INDEX2019 were processed immediately on board or cooled to a temperature of at least − 20 °C until they were processed. In addition, the rolling and ethanol exchange steps recommended by Riehl et al.^18^ were performed. The combination of highly concentrated ethanol and a lower storage temperature is useful to reduce DNA degradation over time^19–21^. All collected specimens were stored at -20 °C.

### DNA extraction

Of the subset of 169 specimens of *Anatoma* used for COI barcoding, a total of 138 specimens was analysed by means of 2b-RAD sequencing: of which 45 specimens being sampled during INDEX2015, 24 specimens during INDEX2018 and 69 specimens during INDEX2019 (Supplementary Table S1). We used the E.Z.N.A.^®^ Mollusc DNA Kit (Omega Bio-tek, Inc., Norcross, GA, USA) to obtain high quality DNA. Specimens were photographed, and the shell opened to allow the enzymes to reach the tissue. DNA was extracted according to the manufacturer’s protocol and the entire specimen, including the shell, was used to obtain the highest possible DNA content.

DNA was measured on the Qubit Fluorometer, using the dsDNA HS (High Sensitivity) Assay Kit (Invitrogen-ThermoFisher Scientific, MA, USA). The amount of DNA required from each specimen was calculated to normalise the concentration to 150 ng DNA in 4.525 µl H_2_O. Therefore, the calculated amount of DNA from each sample was placed on a heat block at 60 °C for 2 h to evaporate the water. If the DNA concentration was too low to measure, the total amount was used.

### 2b-RAD library construction and sequencing

We prepared the 2b-RAD libraries by following the approach developed by Wang et al.^22^. DNA from each sample was digested by adding 0.5 µl of the enzyme BcgI (New England Biolabs, Ipswich, MA, USA), 0.6 µl of 10x NEBuffer 3.1 (New England Biolabs), 4.7125 µl of H_2_O and 0.1875 µl 320 µM SAM (S-adenosylmethionine; New England Biolabs) for 1 h at 37 °C and 20 min at 65 °C. The digested DNA was then ligated in a 26 µl total volume reaction consisting of 0.5 µl 10 nM ATP (New England Biolabs), 1 µl T4 DNA ligase (New England Biolabs), 2 µl 10 x T4 Buffer (New England Biolabs), 14.5 µl H_2_O and 1 µl Adapter R (specific adapters 2–5), 1 µl Adapter F (non-specific adapter) (Adapter information Supplementary Table S2). Finally, 6 µl of digestion product was added and the products were placed on the heat block for 2 h at 25 °C and 20 min at 65 °C.

Each amplification consisted of 8 µl DNA template, 1 µl specific index primer, 0.5 µl each primer (Pri IC1-P5 and Pri IC1-P7) (primer information Supplementary Table S2), 10 µl 2x Phusion Green Hot Start II High Fidelity PCR Master Mix (ThermoFisher Scientific, MA, USA). Cycling conditions of the 2-step PCR were 98 °C for 1 min, 98 °C for 10 s and 72 °C for 15 s (40 cycles), 72 °C for 5 min. The samples were applied to a 2% agarose gel to check the amplification and the target length of the fragments. Then 2 µl of the PCR product were pooled to a new tube (8 specimens per tube). Depending on the strength of the band from previous step, a higher amount of product was used for the pooling (up to 8 µl). To obtain the target products, bands were separated on a 4% agarose gel and further extracted using the Monarch DNA Gel Extraction Kit (New England Biolabs). As a final step, we measured the concentration of each product using the Qubit™ ds DNA Assay Kit and pooled the products for single end sequencing.

The sequenced library consisted of 138 individuals (this study, see Supplementary Table S1) and 103 individuals (another study, another two species) and was first tested on Illumina Miseq using a NanoKit (Illumina) (1 million reads) to check the quality of the runs. Final sequencing was performed on a NextSeq 500 (120 million reads) at the Carl von Ossietzky Universität Oldenburg, Oldenburg, Germany. Raw reads from the Illumina sequencing were deposited in the European Nucleotide Archive (ENA) at EMBL-EBI (accession number: PRJEB63999) https://www.ebi.ac.uk/ena/browser/view/PRJEB63999.

### Genotype calling and filtering

Raw sequencing reads were processed using a custom bash script 2bRADpp downloaded from https://github.com/pmartinezarbizu/2bRADpp. The script uses bbmap for adapter trimming. Reads were oriented in forward direction, PCR duplicates were removed, and reads were demultiplexed by internal barcode.

The remaining data from 125 specimens were processed between and within species, although only the species *A. declivis*, *A. discapex* and *A. laevapex* had enough representatives to be analysed further. Each set of samples (among species, within *A. declivis*, *A. discapex* and *A. laevapex*) was analysed separately using STACKS software version 2.62 ^23,24^. STACKS is a pipeline for building loci from short-read sequences. The algorithm of STACKS reconstructs ‘stacks’ from identical reads from each sample (-m), then either merges them with others to form a single polymorphic locus or keeps them as separate monomorphic loci depending on the number of nucleotide mismatches (-M). We applied the following parameters: -m 8 -M 2 -N 4.

Different scenarios were calculated by POPULATIONS program (within STACKS software version 2.62) to check the influence of the estimated number of populations, as well as the minimum percentage of a read within a population. The two parameters p (minimum number of populations a locus must be present in to process a locus) and r (minimum percentage of individuals in a population required to process a locus for that population) were used for this purpose. The calculated scenarios are: p = 1 and r = 0.1, p = 1 and r = 0.7, p = 2 and r = 0.1, p = 2 and r = 0.7, p = 3 and r = 0.1, p = 3 and r = 0.7. For the between-species analysis, the best approach was to apply parameters for p = 1, r = 0.1 (DS_INMAC_RAD01), and for within-species analysis the more restrictive approach of p = 2, r = 0.1 was used to observe potential differences under strict adjustment (DS_INMAC_RAD02-04). The resulting loci and variant sites of all scenarios can be checked in the supplementary information (Supplementary Table S3).

### Analysis of population structure

The scenarios calculated by POPULATIONS program within STACKS (DS_INMAC_RAD01-04) were further analysed by using the STRUCTURE 2.3.4 ^25^ program. STRUCTURE analyses differences in the distribution of genetic variants amongst populations with a Bayesian iterative algorithm by placing samples into groups (or clusters) whose members share similar patterns of variation. The following parameters were used in the analyses: “admixture model” (assuming that each individual has ancestry from one or more of K genetically distinct sources), correlated allele frequencies, and a burn-in period of 100,000 iterations and 200,000 sampling iterations (following the default settings of Pritchard et al.^26^ ). Analyses were repeated three times for each cluster (k) with a range of 1 to 10 between species and 1 to 5 within species. In addition, the online tool CLUMPAK (Clustering Markov Packager Across K)^27^ was employed for the visualisation of the STRUCTURE plots.

We applied two approaches to calculate the most probable number of clusters K (based on DS_INMAC_RAD01-04, files produced with POPULATIONS): Evanno’s method^28^ using STRUCTURE HARVESTER software version 0.6.94 ^29^ and DAPC (Discriminant Analysis of Principal Components) using the package adegenet version 2.1.5 ^30,31^ in R version 4.2.2^32^ within R STUDIO 2022.12.0 ^33^.

STRUCTURE HARVESTER is a web-based program designed to collate results generated by the program STRUCTURE. It offers a rapid method of assessing and visualising likelihood values across a range of K values and hundreds of iterations, thereby facilitating the identification of the optimal number of genetic groups that align with the dataset. Furthermore, STRUCTURE HARVESTER is capable of reformatting data for utilisation in downstream programs, such as CLUMPAK^29^.

In contrast, the multivariate statistical approach of DAPC partitions the variance in the sample into a between-group and within-group component, thereby optimising discrimination between groups. The genetic data was initially transformed using a PCA (principal component analysis), and clusters are subsequently identified by a DA (discriminant analysis). The DAPC analysis was performed as suggested by Miller et al.^34^ using two different attempts. For the de novo approach, we used the find.clusters function of DAPC to infer the most likely number of clusters. The optimal number of PCs retained was N/3; where N = number of samples as recommended in the manual. The BIC (Bayesian Information Criterion) was calculated and the optimal number of populations with the lowest BIC value was identified. As a second approach, an a priori DAPC analysis was performed using the expected number of clusters (either based on the expected number of species and/or the previously calculated cluster K by Evanno’s method).

Results of the STACKS pipeline (structure files) were used to test hypotheses for analyses of molecular variance (AMOVA). The default method (ade4) was selected to perform an AMOVA by the package poppr^35^ version 2.9.3 in R based on 999 iterations of the three species dataset (DS_INMAC_RAD02, DS_INMAC_RAD03, DS_INMAC_RAD04) to test whether genetic variation was greater (1) between the populations of each vent field, (2) between the samples within one population, or (3) within individuals. For the within individual variance, poppr splits genotypes into haplotypes. Default settings were used.

In addition, the program STRUCTURE calculates the inferred ancestry. Based on this inferred ancestry matrix (of the dataset DS_INMAC_RAD01) we performed an NMDS (Nonmetric Multidimensional Scaling) by using the metaMDS function in the vegan^36^ package version 2.6.2 in R.

The obtained datasets (DS_INMAC_RAD01-04) can be accessed through the Senckenberg Metadata Portal https://dataportal.senckenberg.de/dataset/318754d6-e802-4cb2-a8e5-7f3a4d68af0d.

### Species delimitation

We applied a Bayes Factor Delimitation (*with genomic data; BFD*)^37^ to differentiate species by testing alternative hypotheses of species boundaries based on 2b-RADseq data. The hypotheses were tested against the base scenario (a), which is the current taxonomy as proposed by Hoffman et al.^1^. The following alternative species delimitation models were employed: b) (*A. discapex*) (*A. paucisculpta*) (*Anatoma* sp. 1 DZMB_2021_0095) (*A. declivis* and *A. laevapex*), based on the observation of similarities in their shells, anterior soft parts and radulae^1^ and c) (*A. laevapex*) (*A. paucisculpta*) (*Anatoma* sp. 1 DZMB_2021_0095) (*A. declivis* and *A. discapex*), are similar, as evidenced by their genetic similarity according to the mitochondrial COI data. To ascertain whether the result would differ if fewer sites were excluded, the analysis was repeated with the exclusion of species represented by a limited number of specimens (*A. paucisculpta* and *Anatoma* sp. 1 DZMB_2021_0095).

The data (DS_INMAC_RAD01) produced by POPULATIONS program within STACKS (VCF file) were initially transformed into an XML file by the BEAUti tool, a component of the BEAST 2.6.7^38^ software package. The mutation rates were set to u = 1 and v = 1, and the coalescence rate (population size parameter with one value for each node in the tree) was sampled. This was done while u represented the instantaneous rate of mutating from the ‘0’ allele to the ‘1’ allele and v represented the instantaneous rate of mutating from the ‘1’ allele to the ‘0’ allele. In case of SNP data where the ‘0’ and ‘1’ alleles are arbitrarily assigned from the data, uncoupling these rates is typically not a useful approach^39^. A Γ-distributed prior was employed for the θ parameter (α = 2 and β = 200). To accommodate for uncertainty in the λ parameter (λ refers to the speciation rate in the Yule model), a Γ-distributed hyperprior was applied to this parameter.

Once we established the base scenario in an XML file, we had to edit the file manually for each of the four hypotheses in order to enable the implementation of a path sampling (or stepping stone) analysis. Subsequently, path sampling was performed by utilising the model-selection package, version 1.5.3 in BEAST 2.6.7^38^. A total of 48 steps (1,000 MCMC steps, 0 pre-burnin steps) were employed to estimate marginal likelihoods and species trees for each of the four hypotheses (a, b, c and d). The hypotheses were then ranked in accordance with their estimation of marginal likelihood, and Bayes factors (BF) were calculated to identify the optimal hypothesis for species delimitation, as BF = 2 (ln L_1_ – L_0_). L_0_ and L_1_ represent the estimated marginal likelihoods of the two models under comparison. To assess the significance of the BF, the estimates were employed in compliance with the methodology proposed by Kass and Raftery^40^: 0 < BF < 6 is positive evidence, 6 < BF < 10 is strong support and BF > 10 is decisive.

Furthermore, the XML file was analysed with the SNAPP (SNP and AFLP Package for Phylogenetic analysis) plugin version 1.5.6 implemented in the software BEAST 2 ^38^ for estimating species trees^41^.

### Population genetic metrics

For the three species datasets (DS_INMAC_RAD02-04), the POPULATIONS software in the STACKS pipeline was used to obtain the total number of alleles, number of variant loci (variations of a locus), number of private alleles (found only once in a set of populations), observed heterozygosity (H_O_, observed differing alleles within one gene of one individual), expected heterozygosity (H_E_) under Hardy-Weinberg equilibrium, nucleotide diversity (π), fixation index (F_IS _) and population differentiation (F_ST_). In addition, isolation-by-distance (Mantel test) based on Phi_ST_ (from an AMOVA) was calculated by GENODIVE version 3.06 ^42^.

The genomic datasets were compared with the published mitochondrial COI data (95 sequences) of Hoffman et al.^1^, accessible via the following link: http://www.10.5883/DS-INMAC03. The COI data was analysed by using the DnaSP6 ^43,44^ software by estimating parameters for populations with sample size of *n* ≥ 4 including: gene (u), haplotype (h) and nucleotide (π) diversities^45^, Fu’s Fs^46^ and Tajima’s D^45^.

We estimated minimum spanning networks^47^ to visualise the relationships among the six sampled species based on the published COI dataset by PopART (Population Analysis with Reticulate Trees) (http://popart.otago.ac.nz). We included all sequences from reference libraries in the haplotype network: *Anatoma euglypta* (GenBank accession number: AY923934) from the Antarctic Basin^48^, *A. pseudoequatoria* (MW278816) from the western Pacific Basin, *Anatoma* sp. Lau (AB365210) from the Pacific Lau Basin and *Anatoma* sp. Izu (AB365211) from the northern Pacific Izu Basin^15^. The COI data (95 sequences) published in Hoffman et al.^1^ were updated according to the results of this study and can be downloaded via BOLD (http://www.10.5883/DS-INMAC03).

All data conversions of this study were performed by PGD Spider version 2.1.1.5 ^49^. Distribution map was created by ggOceanMaps^49^ version 2.2.0 in R. Figures were graphically adjusted using Adobe^®^ Photoshop^®^ 25.11.0 software.

## Results

### Raw data filtering

Sequences with average sequencing quality Q < 30 were filtered. All demultiplexed, raw data reads (.fasta) belonging to 138 specimens were checked using the FASTQC High Throughput Sequence Report software (version 0.11.9) and 13 individuals with low number of reads (< 15,000 reads), low number of loci (< 1,500 loci) or abnormal GC content (GC < 50 and > 58) were excluded from further analysis. A total of 125 individuals of *Anatoma* were successfully analysed using 2b-RAD sequencing from six vent fields (VF1, Gauss, VF2, VF3, VF4 and VF5) (Fig. 1) for five different species published by Hoffman et al.^1^: *A. declivis*,* A. discapex*,* A. laevapex*,* A. paucisculpta* and *Anatoma* sp. 1 DZMB_2021_0095. Filtration steps to include loci found in at least 10% of the individuals of one population resulted in 23,856 loci, of which were 12,254 variant (Supplementary Table S3).

### Species delimitation and assignment

**Fig. 2:**
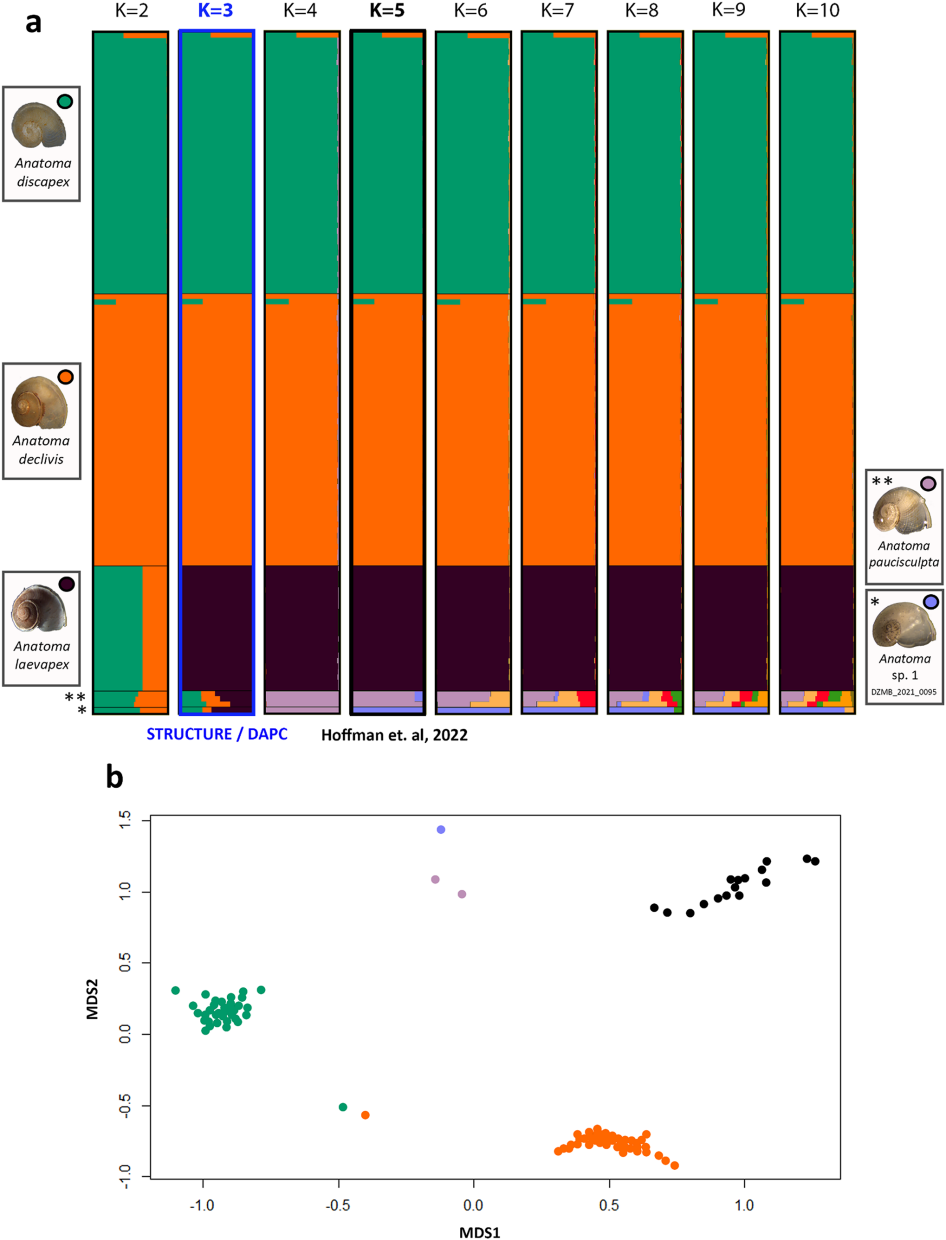
Results of population structure analysis between the species. (a) STRUCTURE assignment based on an admixture model with correlated allele frequencies. The plot indicates the most likely estimated number of clusters K = 3 calculated by STRUCTURE HARVESTER and according to the (de novo) cluster analysis by the package adegenet^30,31^ version 2.1.5 in R . Also highlighted is the expected number of clusters K = 5 according to Hoffman et al.^1^. The barplot was visualized using CLUMPAK. (b) Nonmetric Multidimensional Scaling (NMDS) plot based on the inferred ancestry matrix calculated by STRUCTURE showing the five different species (stress = 0.0868), generated by package vegan^36^ version 2.6.2 in R.

The result of both cluster analyses, DAPC and STRUCTURE HARVESTER, indicated that K = 3 (Supplementary Figures S1 and S2) is the most probable number of clusters K. However, a clear assignment of the specimens to five different clusters was evident in the STRUCTURE barplot (Fig. 2a), which supported the morphological identification and delimitation of species by COI barcoding. The accuracy of the cluster analyses was likely constrained by the underrepresentation of *A. paucisculpta* and *Anatoma* sp. 1 DZMB_2021_0095. Furthermore, an additional 22 specimens for which COI identification was unsuccessful due to DNA degradation were successfully sequenced by 2b-RAD and could consequently be assigned to species.

The species delimitation based on morphology and COI can also be observed in the clustering on the NMDS plot. Five clusters were identified using the genetic ancestry, inferred from the genomic data (Fig. 2b). Two specimens exhibited a signal indicative of potential hybridisation. Both were unambiguously attributed to the species *A. declivis* and *A. discapex* through morphology and COI barcoding.

**Fig. 3:**
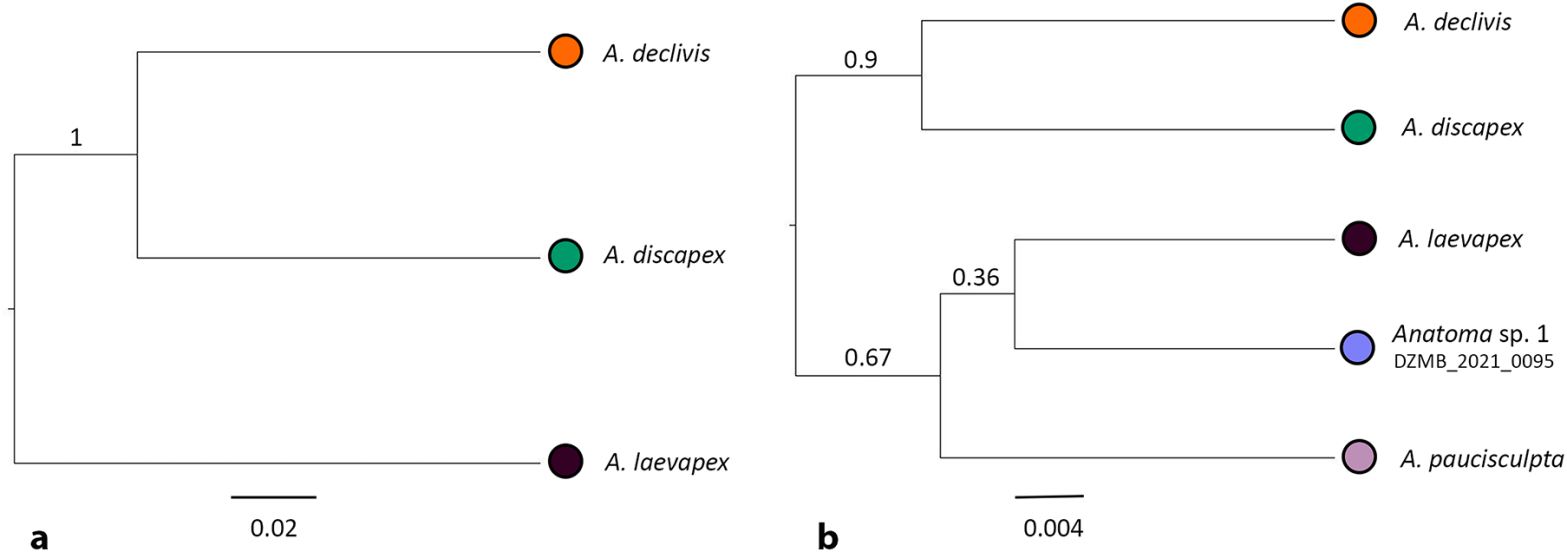
Species tree for the *Anatoma* species estimated with (a) 3115 sites (including the three most abundant species *A. declivis*,* A. discapex* and A. *laevapex*); and (b) 61 sites (including all five species) by using the 2b-RADs dataset and the current taxonomy model (RunA) that separates the species according to the taxonomy done by Hoffman et al.^1^. Posterior probabilities are shown on branches.

In addition, path sampling based on the 2b-RAD data provided further confirmation of the current taxonomy, with full support. The species trees inferred using SNAPP within the software BEAST 2 ^38^ revealed *A. discapex* and *A. declivis* as the most closely related species, for both applied approaches comprising 3115 sites (Supplementary Table S4 and Fig. 3a) and 61 sites (both dataset DS_INMAC_RAD01; Supplementary Table S5 and Fig. 3b).

### *Anatoma* species distribution

**Fig. 4:**
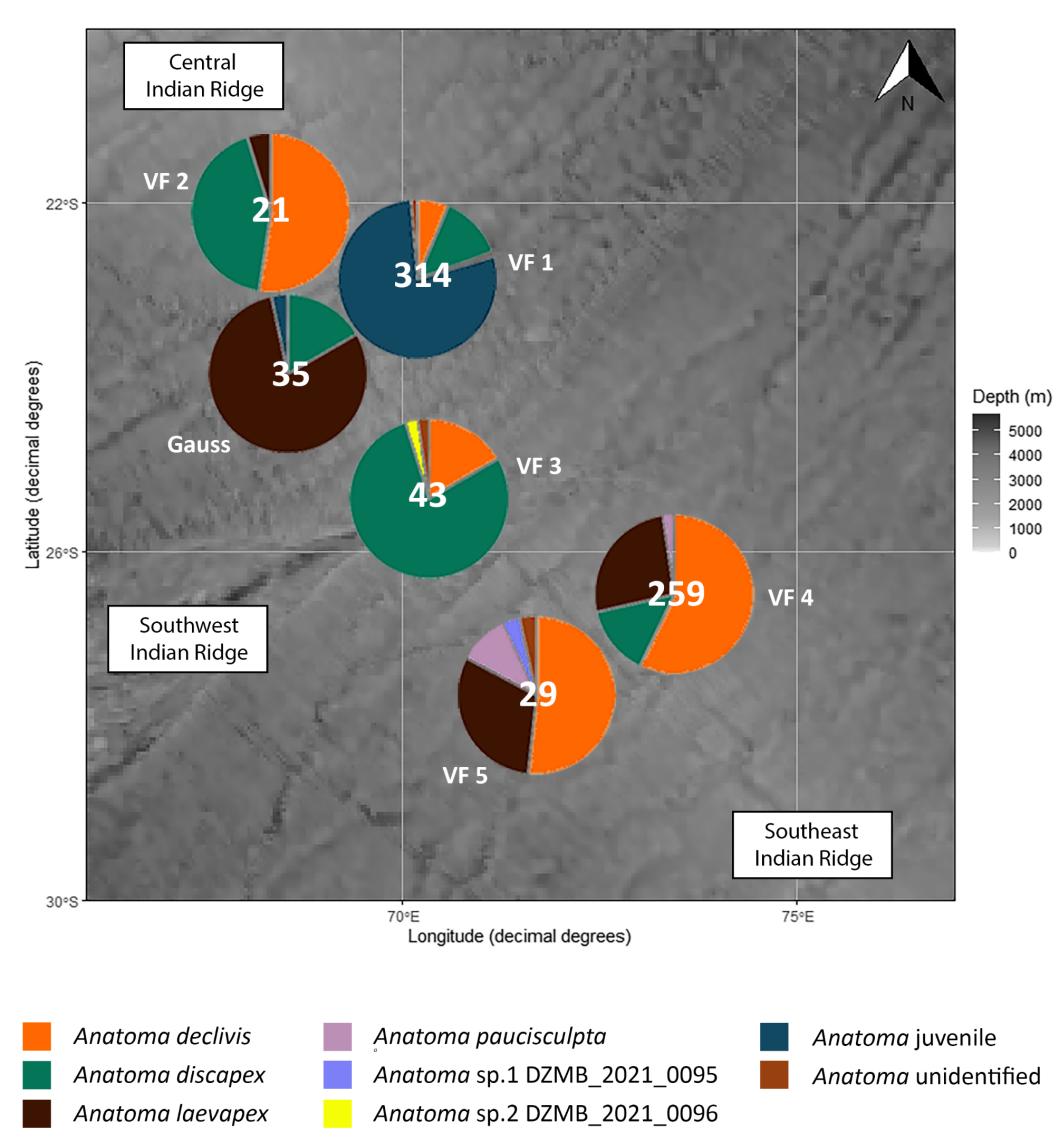
Distribution of the sampled species of *Anatoma* across six vent fields (updated data of Hoffman et al.^1^). Total values are given. Map created by ggOceanMaps^50^ version 2.2.0 in R.

Figure 4 illustrates the updated species distribution within the genus *Anatoma* across the six vent fields. Maximum diversity of the four species was observed in Vent Fields 1, 4 and 5, while Gauss, Vent Field 2 and 3 exhibited three species (Supplementary Table S6). All four species were observed in both the CIR and SEIR. *Anatoma discapex* was documented in five vent fields, *A. declivis* in all six vent fields, *A. laevapex* in five vent fields, and *A. paucisculpta* in only three vent fields. It is evident that the most prevalent species, *A. declivis*, is the most extensively represented.

### Genetic differentiation and population genetic structure

We measured the intraspecific difference for *A. declivis*,* A. discapex* and *A. laevapex* by analysing the RAD data. *Anatoma paucisculpta* and *Anatoma* sp. 1 DZMB_2021_0095 were excluded from the analysis due to the insufficient number of representatives (three individuals of *A. paucisculpta*; one individual of *Anatoma* sp. 1 DZMB_2021_0095).

**Fig. 5:**
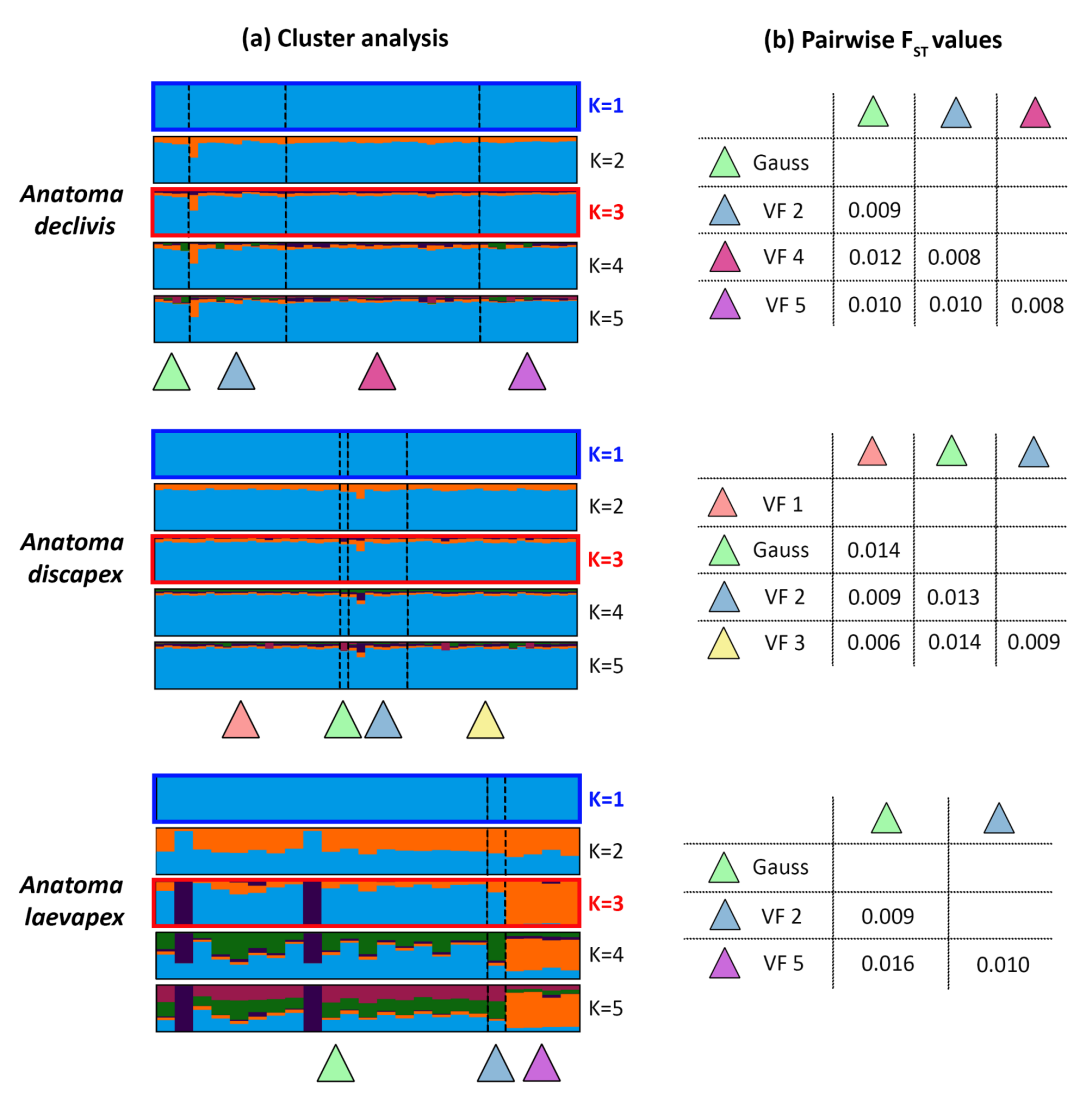
Results for the population analysis of *A. declivis*,* A. discapex* and *A. laevapex*. (a) Cluster analysis of the STRUCTURE calculation (based on an admixture model with correlated allele frequency). The most likely estimated number of clusters K was calculated by STRUCTURE HARVESTER (red) and according to the (de novo) cluster analysis by the package adegenet (blue). The barplot was produced using CLUMPAK. (b) Pairwise F_ST_ matrix was constructed using the sampled vent fields. No pairwise F_ST_ values were significant.

A total of 48 individuals from four vents were studied within the species of *A. declivis*, (Gauss = 4, VF2 = 11, VF4 = 22, VF5 = 11). Following the application of the STACKS analysis, 5,064 loci and 5,148 variant sites were identified as remaining (Supplementary Table S3). The cluster analysis conducted using the STRUCTURE HARVESTER software revealed that the optimum number of clusters K for *A. declivis* was three and DAPC indicated K = 1 (Fig. 5a, Supplementary Figure S3). No evidence for genetic differentiation was observed. The F_ST_ values calculated by STACKS ranged from 0.0077 to 0.0122 and were therefore not statistically significant (Fig. 5b). No species differentiation was observed in the four vent fields in the CIR and SEIR, which corroborated the results of the cluster analysis. For the less distant vent fields, such as Vent Field 4 and 5 (F_ST_ = 0.008) and Vent Field 2 and Gauss (F_ST_ = 0.009) the F_ST_ values were comparatively lower.

We examined 50 individuals of the species *A. discapex* from four vents (VF1 = 22, Gauss = 1, VF2 = 7, VF3 = 20). For *A.* discapex 4,829 loci and 4,617 variant sites were retrieved (Supplementary Table S3), resulting in the optimal number of clusters K = 3 for STRUCTURE HARVESTER and K = 1 for DAPC (Fig. 5a, Supplementary Figure S3). This indicated no evidence of differentiation, as demonstrated by non-significant F_ST_ values ranging from 0.006 to 0.014 (Fig. 5b).

The study of *A. laevapex* encompassed 23 individuals from three distinct vent fields (Gauss = 18, VF2 = 1, VF5 = 4). The cluster analysis included 4,283 loci and 2,207 variant sites (Supplementary Table S3), and yielded the most probable number of clusters K = 3 (STRUCTURE HARVESTER) and K = 1 (DAPC; Fig. 5a, Supplementary Figure S3). The STRUCTURE analysis suggested a genetic differentiation for the population at Vent Field 5; however, the F_ST_ values were not significant (Fig. 5b).

AMOVA showed that the genetic variation is (slightly) lower among samples within populations (− 16.8 − 0.43 %) than among populations (0.4 − 4.1 %), while most of the genetic variation is within individuals (98.5 − 112.7 %) (Table 1). AMOVA statistics among populations resulted in significant p-values, indicating the presence of population structure (Table 1).

**Table 1:** Analysis of Molecular Variance (AMOVA) based on the 2b-RAD dataset showing the partitioning of genetic variation between populations, within populations and within samples for the three species *A. declivis*,* A. discapex* and *A. laevapex*. Table includes source of variation, degree of freedom (df), sum of squares (SS), mean squares (MS) percentage of variation (%) and p-value. Significance calculated by 999 permutations; significant p-values are marked with asterisks (*< 0.05).

Source of variation		Df	SS	MS	%	P
*A. declivis*	Between populations	3	2413.911	804.637	1.048	0.001*
	Between samples within populations	44	28810.623	654.787	0.433	0.608
	Within samples	48	31155.753	649.078	98.518	0.529
*A. discapex*	Between populations	3	1610.349	536.783	0.355	0.054*
	Between samples within populations	46	22879.064	497.371	-5.980	0.966
	Within samples	50	28044.250	560.885	105.625	0.962
*A. laevapex*	Between populations	2	1079.096	539.548	4.134	0.003*
	Between samples within populations	20	7560.648	378.032	-16.786	0.999
	Within samples	23	12385.833	538.515	112.652	0.996

### Population metrics

Genetic distance (Phi_ST_) of the 2b-RAD data (DS_INMAC_RAD02-04) was tested against geographic distance, and a positive correlation was identified. However, the Mantel test for isolation-by-distance yielded a non-significant result (*A. declivis*: r^2^ = 0.609, p = 0.269; *A. discapex*: r^2^ = 0.115, p = 0.603; *A. laevapex*: r^2^ = 0.237, p = 0.666).

The genomic RAD data revealed an overall low heterozygosity for the three species studied across all vent populations and the metapopulations. The F_IS_ value was represented by values close to 0 (Table 2). Additionally, the nucleotide diversity was observed to be low, ranging from 0.00399 to 0.00644 for all loci (variant and fixed). These findings were confirmed by the low nucleotide diversity observed in the COI data, which ranged between π = 0.00473 for *A. discapex* (combined dataset) and π = 0.00481 for *A. declivis* at Gauss (Table 3).

The haplotype diversity calculated based on the COI data was high for *A. discapex* (VF3; h = 0.961), its combined dataset (VF2, Gauss, VF3; h = 0.931) and in *A. declivis* (Gauss; h = 1.000 and the combined set VF1, VF2, Gauss, VF4, VF5; h = 0.829) (Table 3).

The neutrality and population expansion tests yielded negative Tajima’s D values (Table 3), which were found to be statistically significant (p-value < 0.05) for the populations of *A. declivis* (metapopulation and VF4), *A. discapex* (VF3) and for *A. laevapex* (metapopulation and Gauss). The negative D values indicated an excess of rare nucleotides thus an expansion of the populations. This hypothesis was supported by the negative Fu’s Fs values (Table 3).

**Fig. 6:**
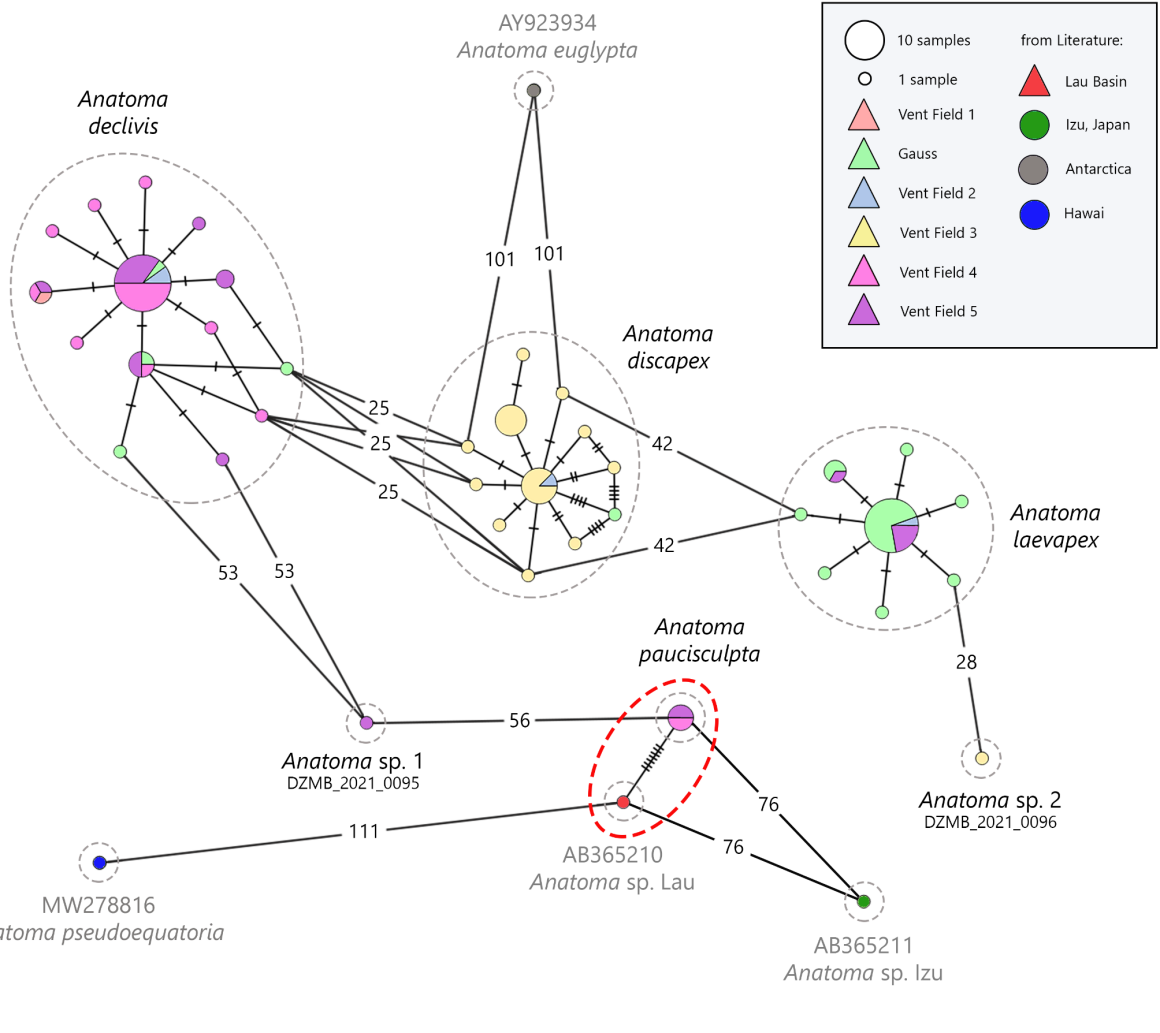
Results of the statistical haplotype network analysis conducted using the PopART program on the COI dataset, including all *Anatoma* species from GenBank. The analysis is based on an alignment of 658 bp. The colours correspond to the different sampling sites and the size of each circle is proportional to the number of individuals (see legend). Hatch marks indicate single substitutions. Mutations (n > 10) are presented as numbers. The triangles in the legend refer to hydrothermal vents at the sampling locality. The high degree of genetic similarity between *A. paucisculpta* and *Anatoma* sp. Lau is indicated with a red circle.

The number of observed haplotypes varied from 1 to 19 (Table 3; Fig. 6). Based on the haplotype network (Fig. 6) a distinct separation between the six species with a mutation rate of 25 to 56 substitutions was demonstrated. The minimum intra-specific difference was observed between *A. declivis* and *A. discapex* (25 substitutions). The corresponding mean intra-specific difference was notably smaller than the inter-specific difference. These two species were therefore conclusively separated.

**Table 2:** Summary genetic statistics of each local population for all positions (variant and fixed) and for variant positions only, which are present in two populations and in 10% of the individuals (datasets DS_INMAC_RAD02-04); variant sites, number of unique SNPs (private), polymorphic sites, expected heterozygosity (H_E_), observed heterozygosity (H_O_), nucleotide diversity (π) and inbreeding coefficient (F_IS_). Combined populations (= metapopulation) of the six locations are presented as “meta”.

All positions – variant and fixed									
Species	Pop	*N*	Private	Variant sites	Polymorphic sites	H_E_	H_O_	π	F_IS_
*A. declivis*	meta	48	2590	8651	5247	0.00587	0.00513	0.00605	0.00437
	Gauss	4	165	4770	1659	0.00457	0.00539	0.00644	0.00085
	VF 2	11	754	4947	3026	0.00584	0.00581	0.00611	0.00178
	VF 4	22	1075	5059	3695	0.0058	0.00539	0.00585	0.00278
	VF 5	11	314	4434	2238	0.00533	0.00543	0.0059	0.0014
*A. discapex*	meta	50	2147	8363	4572	0.00525	0.00479	0.00542	0.00339
	VF 1	22	835	4291	3050	0.00548	0.00533	0.00585	0.00207
	Gauss	1	37	2571	384	0.00298	0.00595	0.00579	0
	VF 2	7	409	4530	2170	0.00476	0.00569	0.00573	0.00045
	VF 3	20	841	4484	3067	0.00532	0.00522	0.00595	0.002
*A. laevapex*	meta	23	963	4987	2157	0.00377	0.00415	0.00399	0.0002
	Gauss	18	863	2180	1933	0.00415	0.00477	0.00438	-0.0003
	VF 2	1	55	1855	511	0.00219	0.00438	0.00444	0
	VF 5	4	192	2127	1115	0.00343	0.00461	0.00449	-0.0002

Variant positions									
**Species**	**Pop**	**N**	**Private**			**H**_**E**_	**H**_**O**_	**π**	**F**_**IS**_
*A. declivis*	meta	48	2590			0.11492	0.10042	0.11843	0.08541
	Gauss	4	165			0.16824	0.16721	0.18544	0.05123
	VF 2	11	754			0.17381	0.16147	0.18294	0.08319
	VF 4	22	1075			0.13065	0.15407	0.16724	0.02434
	VF 5	11	314			0.14885	0.1516	0.1648	0.03909
*A. discapex*	meta	50	2147			0.10042	0.09155	0.10363	0.06489
	VF 1	22	835			0.15583	0.18649	0.19157	0.01466
	Gauss	1	37			0.16433	0.15986	0.17364	0.06212
	VF 2	7	409			0.17105	0.16778	0.18431	0.06427
	VF 3	20	841			0.07468	0.14936	0.14936	0
*A. laevapex*	meta	23	963			0.11504	0.12663	0.12161	0.0062
	Gauss	18	863			0.13774	0.27547	0.27547	0
	VF 2	1	55			0.24777	0.28449	0.26521	-0.01808
	VF 5	4	192			0.20905	0.28126	0.27381	-0.01219

**Table 3:** Genetic diversity indices, parameters of demographic history and neutrality and population expansion tests calculated for the dataset based on COI barcodes of *Anatoma* species in the central Indian Ocean. Significant p-values are marked with asterisks (*< 0.05). Only species and populations represented by a total n ≥ 4 are shown. Combined populations (= metapopulation) of the six locations are presented as “meta”. Unpublished vent fields are abbreviated as “VF “.

Species	Location	n	No. of haplotypes	Haplotype diversity (h)	Nucleotide diversity (π)	Tajima’s D	Fu’s Fs
*A. declivis*	Gauss	4	4	1.000	0.00481	-0.31446	-1.157
	VF 4	18	10	0.765	0.00205	-1.85426*	-8.672
	VF 5	14	9	0.835	0.00262	-0.89259	-6.535
	meta	39	19	0.829	0.00281	-1.86482*	-19.264
*A. discapex*	VF 3	22	16	0.961	0.00476	-1.86463*	-12.778
	meta	24	14	0.931	0.00473	-1.61259	-8.755
*A. laevapex*	Gauss	21	9	0.681	0.00169	-2.05736*	-7.467
	VF 5	4	3	0.833	0.00195	-0.70990	-0.887
	meta	26	8	0.572	0.00145	-1.93660*	-6.202
*A. paucisculpta*	meta	4	1	-	-	-	-

## Discussion

### RAD sequencing - suitable method for species delimitation

By assigning additional specimens to species this study demonstrates that 2b-RAD sequencing is a more suitable method for degraded DNA^51,52^ compared to the COI marker. The cytochrome c oxidase I (COI) gene, which is around 650 basepairs (bp) in length and located within the mitochondrial genome, is a useful DNA barcode to provide signals of population history over short periods of time due to its relatively high mutation rate^53^. In addition, to gain insights into the population over longer time frames, it is necessary to examine the nuclear DNA. The assessment of the entire nuclear genome is expensive, especially when comparing several specimens from different populations. By contrast, 2b-RAD sequencing is a whole-genome sequencing method that examines multiple loci through the analysis of short fragments of 32–35 bp.

### High similarity to other species from hydrothermal vent fields – are the species vent-endemic?

Based on COI analyses, our studied anatomids were genetically similar to *Anatoma* sp. Lau, a specimen obtained from hydrothermal vent fields in the Lau Basin (South Pacific Ocean) at a depth of 1817 m^15^ (Fig. 6). The distance between the Lau Basin and the Rodriguez Triple Junction (RTJ) is approximately 11,200 km. The three other species for which a COI barcode was available exhibited a greater genetic distance from the species under this study (Fig. 6): *Anatoma euglypta* (Pelseneer, 1903) from Pine Island Bay, Amundsen Sea, Antarctica^48^ (approx. 4,700 km from the RTJ), *A. pseudoequatoria* (Kay, 1979) from reef and shore in Hawaii (unpublished, approx. 15,600 km from the RTJ) and *Anatoma* sp. Izu from intertidal zones Izu, Shizuoka, Japan^15^ (approx. 10,200 km from the RTJ). It can therefore be concluded that *A. euglypta* and *Anatoma* sp. Izu are geographically less distant from our species, which indicates that anatomids inhabiting vent ecosystems may be genetically closer to one another than non-vent species.

The INDEX project encompasses the study of the seafloor at hydrothermal vent fields and their surrounding habitats. It offers a distinctive opportunity for annual sampling with an ROV, which produced a substantial amount of data, including video footage, seafloor maps, and information on the water masses. In addition, a total of 20,000 macrofauna specimens were collected from hydrothermal vents and surrounding non-vent seafloors. Therefore, we can conclude, that the anatomid species analysed in this study were exclusively sampled in the immediate vicinity of vents (active and inactive hydrothermal vent areas) and not even one in non-vent habitats (personal observation). This leads us to the assumption that the anatomids are only associated with hydrothermal vents.

The combination of the occurrence of our sampled anatomids exclusively in chemosynthetically active environments and the high similarity to the specimen *Anatoma* sp. Lau leads us to the hypothesis, that our species are probably vent-endemic. This term is used to describe species that are restricted to an ecosystem rather than to a specific location^54^. The feeding traits of our species may also enhance the likelihood of endemism, as they feed most probably on the present bacterial mats^1^.

### *Anatoma* populations indicate geneflow along the CIR and SEIR

2b-RAD sequencing was employed to evaluate the population structure of the three most prevalent anatomid species along the CIR and SEIR, *A. declivis*,* A. discapex* and *A. laevapex*. The results demonstrated that these species exhibited identical population patterns, characterised by panmictic populations and the absence of genetic differentiation across the entire ridge system (Table 1; Figs. 5 and 6). Although the AMOVA results indicated weak but significant population structure among populations (Table 1), this contrasted with non-significant F_ST_ values and non-significant Mantel test for isolation-by-distance. Highly connected populations are the result of most of the connectivity studies along the CIR^9–12,55^. This study underscores the well-connected vent populations along the entire ridge system, despite the patchy and dispersed nature of their habitats.

Furthermore, it indicates that species of *Anatoma* are capable of dispersing between vent fields over a distance of approximately 800 km, which can be explained by the present water masses. The sampled vent fields in this study are influenced by the Circumpolar Deep Water (CDW), which ranges in depth from 2,000 to 2,500 m (see Fig. 6 in Harms et al.^56^). The Indian Deep Water (IDW), with a flow direction from north to south and a depth range of 2,000–1,250 m, is present above the CDW. In addition, a complex sequence of water masses is known to exist, characterised by a variety of current directions and properties^56^. If larvae can enter the currents of these different water masses, the studied species may disperse in any direction.

Moreover, an extended duration in a planktonic phase will serve to enhance dispersion. Many vent invertebrate species exhibit lecithotrophic larval development, which is characterised by a dependency on energy reserves stored in the yolk. Nevertheless, this results in a particular larval phase^57^. Alternatively, there may be a lecithotrophic larval stage for at least part of their development, followed by a plankton-based diet in subsequent stages^8^. In contrast, it has been proposed that planktotrophic larvae have considerable potential for dispersal^6^. The gastropod (genera suggested: *Lepetodrilus* & *Phymorhynchus*) and bivalve larvae (genus suggested: *Bathymodiolus*) at Solitaire and Onnuri Vent Field on the CIR were sampled in the upper layers of the water column (0–200 m), indicating that these larvae may disperse approximately 2,000 m above the vents^58^. The larval shells of the sampled *Anatoma* indicate either a direct development with large (and hence few) eggs or a short lecithotrophic phase^1^. To the best of our knowledge, anatomid larvae *have not yet been sampled* in plankton samples.

It seems reasonable to posit that transform faults may act as an impassable barrier for veliger larvae of numerous gastropods, given that these larvae are inclined to remain in the proximity of the seabed^59^. An example of low gastropod species connectivity in the Indian Ocean is *Chrysomallon squamiferum*, which is endemic to the central Indian Ocean vent fields^11^. Significant genetic differentiation is observed between the southern SWIR and CIR, as well as between the CIR and CR^13,14^. This differentiation can likely be attributed to transform faults. Furthermore, differentiation is observed between the southern SWIR and CIR for two additional species (*Neoplepas marisindica* and *Bathymodiolus septemdierum*), and between the CIR and CR for *Neoplepas marisindica*, *Chrysomallon squamiferum*, *Bathymodiolus septemdierum* and *Hesiolyra heteropoda*^14^. To determine whether these “differentiation-zones” are present within *Anatoma*, further sampling on the CR and SWIR is required.

### Expanding populations reveal small gene pool, hence a vulnerability to mining activities

In addition to the high connectivity among the populations of *Anatoma*, Tajima’s D and Fu’s Fs calculations on the COI data revealed expanding populations (Table 3). This phenomenon is commonly observed in hydrothermal vent populations^60–64^. This indicates that the populations may be relatively young sharing a recent common history of bottleneck or founder events, and expansion. This assumption is confirmed by the 2b-RAD data, as the observed heterozygosity (H_O_) was slightly lower than the expected heterozygosity (H_E_), suggestive of an earlier population bottleneck.

We observed extremely low heterozygosity (H_0_ = 0.09155–0.28449; for variant sites) (Table 2), indicating that there is minimal genetic variability. Similarly low values have been documented for the vent species *Bathymodiolus platifrons* (H_O_ = 0.1480–0.1633)^65^. In contrast, the values for microsatellite loci for *Bathymodiolus manusensis* from the Manus Basin were considerably higher (H_O_ = 0.24–0.94)^62^, with similar results observed by Teixeira et al.^66^ for *Rimicaris exoculata* (H_O_ = 0.58–0.68). In the case of *Munidopsis lauensis* and *Chorocaris* sp. 2, the calculated H_O_ ranges are larger^61^.

Coykendall et al.^67^ establish a correlation between H_E_ of *Riftia pachyptila* (ranging from approximately H_E_ = 0.1 to 0.5) and tectonic spreading rates. Their findings indicate that lower heterozygosity is associated with faster spreading rates. In our case, however, this theory cannot be confirmed. The CIR is a slow- to intermediate-spreading ridge^68^, whereas the SEIR is an intermediate- to fast-spreading ridge^69^. No significant difference was observed in heterozygosity between the CIR and SEIR.

The NMDS plot (Fig. 2b) from the 2b-RAD dataset indicated the potential for hybridisation between *A. declivis* and *A. discapex* in two specimens. On the basis of mitochondrial and morphological characteristics they were unambiguously assigned to the respective species as either *A. declivis* or *A. discapex*. Hybridisation is a common phenomenon in Gastropoda, and is detected at vent fields within mussel species of *Bathymodiolus*^70–72^, respectively. Further sampling and analyses will be necessary to support this theory and to estimate the effects that hybridisation will have regarding the formation of new species within *Anatoma*.

Although connectivity was high for all three species, low heterozygosity indicated a small gene pool and therefore, high sensitivity to environmental change. Serious damage to hydrothermal vent ecosystems is predicted from future deep-sea mining or other anthropogenic impacts^73^. To ascertain whether the unique vent field fauna can be conserved through the establishment of marine protected areas, further research on genetic connectivity is required to develop a conservation plan also for other species in this ecological niche.

### Indian Ocean functions as dispersal corridor for hydrothermal vent species

Several vent-endemic genera occur in Atlantic, Pacific and Indian Ocean, such as *Bathymodiolus*, *Munidopsis* and *Phymorhynchus*^74^, whereas shared taxa between Atlantic and Pacific Ocean vents are leading to the theory that the Indian Ocean serves as a dispersal conduit between ocean basins^74,75^. The similarity of *A. paucisculpta* from *Anatoma* sp. Lau is supporting this theory. Hoffman et al.^1^ distinguishes them by four out of six delimitation methods. Haplotype analyses revealed only seven mutations between *Anatoma* sp. Lau and *A. paucisculpta* (Fig. 6).

A similar pattern is observed by Hwang et al.^76^ for the squat lobster *Munidopsis lauensis* from specimens collected from the Onnuri Vent Field (Indian Ocean), the Brothers Seamount, Manus, and Lau Basins (both southwest Pacific Ocean). The evolution of hydrothermal species from shallow specimens and a dispersion corridor is hypothesized. Besides, their results indicate that the western Pacific population diverged before the Indian Ocean population^76^.

### Species occurrence on small spatial scale suggests sympatric speciation event

In addition, the genetic similarity between *A. paucisculpta* from *Anatoma* sp. Lau suggests the possibility of a recently, geographically diverged species. Anatomids in the Indo-Pacific exhibit a wide distribution, with some species extending over 10,000 km^77^. Only two anatomids were previously reported by Geiger^77^ to inhabit depths exceeding 2,000 m in the Indian Ocean. However, the recent additions by Hoffman et al.^1^ suggest that additional species may occupy lower bathyal and abyssal depths.

A broad distribution was evident for *A. declivis*,* A. discapex*, *A. laevapex* and *A. paucisculpta* along the ridge systems in the Indian Ocean and it is likely that some species were not sampled at certain vents due to either undersampling or unsuitable environmental conditions for the species (environmental filtering). The present study demonstrated the co-existence of six *Anatoma* species at the small spatial scale, with two to three species observed on a single sampled small rock or piece of chimney, and four to five species sampled within a radius of less than ten metres. The speciation of *Anatoma* at Indian Ocean vent fields may be attributed to the presence of diverse bacterial mats^78,79^, which serve as a potential food source for different anatomid species^1^. The presence of the two single specimens in this study suggests that there are likely numerous additional species of *Anatoma* at the hydrothermal fields in the Indian Ocean, that have not been sampled due to their occurrence in smaller numbers.

Finally, it must be mentioned, in addition to offering a lot of new insights, this study also presents certain limitations, such as the small number of specimens from some locations. Population genetic studies typically require at least ten individuals per sampling site to ensure accurate results^80^. Nevertheless, this study makes a significant contribution to the field of connectivity studies along the Indian Ocean ridge system, as there have been no previous studies on population connectivity at the SEIR. The findings of this study indicate that *Anatoma* populations exhibit no discernible differentiation along the investigated trajectories across the CIR and SEIR. Moreover, this study addresses numerous questions pertaining to the distribution and global connectivity of the Anatomidae. To enhance comprehension of anatomid populations at hydrothermal vents, it is imperative to augment the COI dataset with genetic material from the CR, SWIR, Pacific, and Atlantic vent species, complemented by genomic data. Additionally, the scarcity of the species *A. paucisculpta* and the two single specimens necessitates further sampling to elucidate their role at Indian Ocean hydrothermal vents.

## Conclusions

2b-RAD is a suitable method for species delimitation in addition to the traditional species differentiation by morphology and the molecular identification by COI. Many of the short DNA fragments analysed by 2b-RAD provide superior determinations when the genetic code is degraded. Long-distance connectivity of vent anatomids is indicated by the high similarity between *A. paucisculpta* and a specimen from a hydrothermal vent in the Pacific Ocean. Furthermore, this study confirms the theory of a dispersal corridor between the western Pacific and the Indian Ocean. Three species *A. declivis*,* A. discapex* and *A. laevapex* show a high genetic similarity over approximately 800 km, revealing a high gene flow and good connectivity between the vent fields. However, low heterozygosity highlights the vulnerability of the present fauna towards catastrophic events due to limited genetic diversity. Finally, this study demonstrates, how 2b-RAD sequencing in combination with other molecular methods can provide important information on population structure and dynamics in hydrothermal vent ecosystems, which is necessary for the conservation and management of these ecosystems with regards to potential future mining.

## Electronic supplementary material

Below is the link to the electronic supplementary material.

Supplementary Material 1

## Data Availability

The 2b-RADs raw reads generated during the current study have been deposited in the European Nucleotide Archive (ENA) at EMBL-EBI under accession number PRJEB63999 (https://www.ebi.ac.uk/ena/browser/view/PRJEB63999) and the obtained datasets can be accessed through the Senckenberg Metadata Portal https://dataportal.senckenberg.de/dataset/318754d6-e802-4cb2-a8e5-7f3a4d68af0d. The COI data of Hoffman et al.^1^ was updated and is available in BOLD (10.5883/DS-INMAC03).
