# Supplementary material for: High genomic connectivity within Anatoma at hydrothermal vents along the Central and Southeast Indian Ridge
Source: Sci Rep. 2025 Jan 15;15:1971. doi: 10.1038/s41598-025-85507-z (PMC11732982; doi:10.1038/s41598-025-85507-z)
Supplement: Supplementary file 1 — Supplementary Material 1 [file 41598_2025_85507_MOESM1_ESM.docx]

**High genomic connectivity within *Anatoma* at hydrothermal vents along the Central and Southeast Indian Ridge - Supplementary information**

Katharina Kniesz ^1, 2, 3 *^, Leon Hoffman ^1^, Pedro Martínez Arbizu ^1, 2, 4^, and Terue C. Kihara ^4^

^1^ Senckenberg am Meer, Wilhelmshaven, Germany

^2^ Carl von Ossietzky Universität Oldenburg, Oldenburg, Germany

^3^ Leibniz-Institut für Ostseeforschung Warnemünde, Rostock, Germany

^4^ INES Integrated Environmental Solutions UG, Wilhelmshaven, Germany

Table S1: List of all anatomid samples (updated table of Hoffman et al., 2022, according to this study) with information about sampling locations including sampling date, station, vent field (VF), coordinates, depth, sample type and sampling tool. All studied anatomids and haplo-/paratype material with voucher specimen code, species name, total number, BOLD process ID, GenBank accession number, museum ID and information about RADseq (successfully sequenced: Y, successfully sequenced + assigned to species by RADseq: Y+, not successfully sequenced and excluded: Ex).

| **^INDEX cruise^** | **^Sampling date^** | **^Station^** | **^Sample ID^** | **^Vent Field^** | **^Latitude^** | **^Longitude^** | **^Depth (m)^** | **^Sample type^** | **^Sampling tool^** | **^Voucher Specimen Code^** | **^Species^** | **^No. specimen^** | | **^Process ID^** | | **^GenBank accession number^** | | **^Museum ID^** | | **^RADs (Y/Y+/Ex)^** | |
| --- | --- | --- | --- | --- | --- | --- | --- | --- | --- | --- | --- | --- | --- | --- | --- | --- | --- | --- | --- | --- | --- |
| ^INDEX2015^ | ^02.12.2015^ | ^I15_49R^ | ^I15_49R_F_8^ | ^VF 2^ | ^23.78°S^ | ^69.55°E^ | ^3048.0^ | ^rock^ | ^claw^ | ^I15_Ma_141^ | *^A. discapex^* | ^1^ | ^INMAC319-21^ | | ^-^ | | ^-^ | | ^Y+^ | |  |
|  |  |  |  |  |  |  |  |  |  | ^I15_Ma_142^ | *^A. discapex^* | ^1^ | ^INMAC320-21^ | | ^-^ | | ^-^ | | ^Y+^ | |  |
|  |  |  |  |  |  |  |  |  |  | ^I15_Ma_143^ | *^A. discapex^* | ^1^ | ^INMAC321-21^ | | ^-^ | | ^-^ | | ^Y+^ | |  |
|  |  |  |  |  |  |  |  |  |  | ^I15_Ma_144^ | *^A. discapex^* | ^1^ | ^INMAC322-21^ | | ^OM951082^ | | ^-^ | | ^Y^ | |  |
|  |  |  | ^I15_49R_B1_1^ | ^VF 2^ | ^23.78°S^ | ^69.55°E^ | ^3049.0^ | ^rock^ | ^claw^ | ^I15_Ma_2^ | *^A. laevapex^* | ^1^ | ^INMAC302-21^ | | ^OM951097^ | | ^-^ | | ^Y^ | |  |
|  |  |  | ^I15_49R_B2_1^ | ^VF 2^ | ^23.78°S^ | ^69.55°E^ | ^3049.0^ | ^rock^ | ^claw^ | ^I15_Ma_8^ | *^A. discapex^* | ^1^ | ^INMAC303-21^ | | ^OM951025^ | | ^-^ | | ^Ex^ | |  |
|  |  |  |  |  |  |  |  |  |  | ^I15_Ma_9^ | *^A. declivis^* | ^1^ | ^INMAC304-21^ | | ^OM951051^ | | ^-^ | | ^Y^ | |  |
|  |  |  |  |  |  |  |  |  |  | ^I15_Ma_128^ | *^A. declivis^* | ^1^ | ^INMAC305-21^ | | ^OM951114^ | | ^-^ | | ^Y^ | |  |
|  |  |  |  |  |  |  |  |  |  | ^I15_Ma_129^ | *^A. declivis^* | ^1^ | ^INMAC306-21^ | | ^OM951024^ | | ^-^ | | ^Y^ | |  |
|  |  |  |  |  |  |  |  |  |  | ^I15_Ma_130^ | *^A. declivis^* | ^1^ | ^INMAC307-21^ | | ^OM951122^ | | ^-^ | | ^Y^ | |  |
|  |  |  |  |  |  |  |  |  |  | ^I15_Ma_131^ | *^A. declivis^* | ^1^ | ^INMAC308-21^ | | ^-^ | | ^-^ | | ^Y^ | |  |
|  |  |  |  |  |  |  |  |  |  | ^I15_Ma_132^ | *^A. declivis^* | ^1^ | ^INMAC309-21^ | | ^-^ | | ^-^ | | ^Y+^ | |  |
|  |  |  |  |  |  |  |  |  |  | ^I15_Ma_133^ | *^A. discapex^* | ^1^ | ^INMAC310-21^ | | ^-^ | | ^-^ | | ^Ex^ | |  |
|  |  |  |  |  |  |  |  |  |  | ^I15_Ma_134^ | *^A. declivis^* | ^1^ | ^INMAC311-21^ | | ^OM951089^ | | ^-^ | | ^Y^ | |  |
|  |  |  | ^I15_49R_B2_2^ | ^VF 2^ | ^23.78°S^ | ^69.55°E^ | ^3049.0^ | ^rock^ | ^claw^ | ^I15_Ma_32^ | *^A. declivis^* | ^1^ | ^INMAC312-21^ | | ^OM951127^ | | ^-^ | | ^Y^ | |  |
|  |  |  |  |  |  |  |  |  |  | ^I15_Ma_135^ | *^A. discapex^* | ^1^ | ^INMAC313-21^ | | ^OM951105^ | | ^-^ | | ^Y^ | |  |
|  |  |  |  |  |  |  |  |  |  | ^I15_Ma_136^ | *^A. discapex^* | ^1^ | ^INMAC314-21^ | | ^OM951035^ | | ^-^ | | ^Y^ | |  |
|  |  |  |  |  |  |  |  |  |  | ^I15_Ma_137^ | *^A. declivis^* | ^1^ | ^INMAC315-21^ | | ^OM951034^ | | ^-^ | | ^Y^ | |  |
|  |  |  |  |  |  |  |  |  |  | ^I15_Ma_138^ | *^A. declivis^* | ^1^ | ^INMAC316-21^ | | ^OM951058^ | | ^-^ | | ^Y^ | |  |
|  |  |  |  |  |  |  |  |  |  | ^I15_Ma_139^ | *^A. discapex^* | ^1^ | ^INMAC317-21^ | | ^-^ | | ^-^ | | ^Y+^ | |  |
|  |  |  | ^I15_49R_B3_2^ | ^VF 2^ | ^23.78°S^ | ^69.55°E^ | ^3049.0^ | ^rock^ | ^claw^ | ^I15_Ma_140^ | *^A. declivis^* | ^1^ | ^INMAC318-21^ | | ^-^ | | ^-^ | | ^Y+^ | |  |
|  | ^04.12.2015^ | ^I15_53R^ | ^I15_53R_A_2^ | ^VF 1^ | ^23.88°S^ | ^69.62°E^ | ^3171.0^ | ^rock^ | ^claw^ | ^-^ | *^A. laevapex^* | ^2^ | ^-^ | | ^-^ | | ^-^ | | ^-^ | |  |
|  | ^08.12.2015^ | ^I15_62R^ | ^I15_62R_D_1^ | ^VF 1^ | ^23.88°S^ | ^69.62°E^ | ^3296.0^ | ^rock^ | ^claw^ | ^I15_Ma_145^ | *^A. discapex^* | ^1^ | ^INMAC323-21^ | | ^OM951103^ | | ^-^ | | ^Y^ | |  |
|  |  |  |  |  |  |  |  |  |  | ^I15_Ma_146^ | ^unidentified^ | ^1^ | ^INMAC324-21^ | | ^-^ | | ^-^ | | ^Ex^ | |  |
|  |  |  |  |  |  |  |  |  |  | ^I15_Ma_147^ | *^A. discapex^* | ^1^ | ^INMAC325-21^ | | ^OM951123^ | | ^-^ | | ^Y^ | |  |
|  |  |  |  |  |  |  |  |  |  | ^I15_Ma_169^ | *^A. paucisculpta^* | ^1^ | ^INMAC445-21^ | | ^-^ | | ^SMF 358992^ | | ^-^ | |  |
|  |  |  |  |  |  |  |  |  |  | ^-^ | *^A. discapex^* | ^8^ | ^-^ | | ^-^ | | ^-^ | | ^-^ | |  |
|  |  |  | ^I15_62R_D_2^ | ^VF 1^ | ^23.88°S^ | ^69.62°E^ | ^3296.0^ | ^rock^ | ^claw^ | ^I15_Ma_148^ | *^A. discapex^* | ^1^ | ^INMAC326-21^ | | ^OM951063^ | | ^-^ | | ^Y^ | |  |
|  |  |  |  |  |  |  |  |  |  | ^I15_Ma_149^ | *^A. discapex^* | ^1^ | ^INMAC327-21^ | | ^OM951111^ | | ^-^ | | ^Y^ | |  |
|  |  |  |  |  |  |  |  |  |  | ^I15_Ma_150^ | *^A. discapex^* | ^1^ | ^INMAC328-21^ | | ^OM951036^ | | ^-^ | | ^Y^ | |  |
|  |  |  |  |  |  |  |  |  |  | ^I15_Ma_151^ | *^A. discapex^* | ^1^ | ^INMAC329-21^ | | ^-^ | | ^-^ | | ^Y+^ | |  |
|  |  |  |  |  |  |  |  |  |  | ^I15_Ma_152^ | *^A. discapex^* | ^1^ | ^INMAC330-21^ | | ^-^ | | ^-^ | | ^Y+^ | |  |
|  |  |  |  |  |  |  |  |  |  | ^I15_Ma_153^ | *^A. discapex^* | ^1^ | ^INMAC331-21^ | | ^-^ | | ^-^ | | ^Y+^ | |  |
|  |  |  |  |  |  |  |  |  |  | ^I15_Ma_154^ | *^A. discapex^* | ^1^ | ^INMAC332-21^ | | ^OM951104^ | | ^-^ | | ^Y^ | |  |
|  |  |  |  |  |  |  |  |  |  | ^I15_Ma_155^ | *^A. discapex^* | ^1^ | ^INMAC333-21^ | | ^-^ | | ^-^ | | ^Y+^ | |  |
|  |  |  |  |  |  |  |  |  |  | ^I15_Ma_156^ | *^A. discapex^* | ^1^ | ^INMAC334-21^ | | ^-^ | | ^-^ | | ^Y+^ | |  |
|  |  |  |  |  |  |  |  |  |  | ^I15_Ma_157^ | *^A. discapex^* | ^1^ | ^INMAC335-21^ | | ^-^ | | ^-^ | | ^Y+^ | |  |
|  |  |  |  |  |  |  |  |  |  | ^I15_Ma_158^ | *^A. discapex^* | ^1^ | ^INMAC336-21^ | | ^-^ | | ^-^ | | ^Y^ | |  |
|  |  |  |  |  |  |  |  |  |  | ^I15_Ma_159^ | *^A. discapex^* | ^1^ | ^INMAC337-21^ | | ^-^ | | ^-^ | | ^Y+^ | |  |
|  |  |  |  |  |  |  |  |  |  | ^I15_Ma_160^ | *^A. discapex^* | ^1^ | ^INMAC338-21^ | | ^-^ | | ^-^ | | ^Y+^ | |  |
|  |  |  |  |  |  |  |  |  |  | ^I15_Ma_161^ | *^A. discapex^* | ^1^ | ^INMAC339-21^ | | ^-^ | | ^-^ | | ^Y+^ | |  |
|  |  |  |  |  |  |  |  |  |  | ^I15_Ma_162^ | *^A. discapex^* | ^1^ | ^INMAC340-21^ | | ^-^ | | ^-^ | | ^Y+^ | |  |
|  |  |  |  |  |  |  |  |  |  | ^I15_Ma_163^ | *^A. discapex^* | ^1^ | ^INMAC341-21^ | | ^OM951095^ | | ^-^ | | ^Y^ | |  |
|  |  |  |  |  |  |  |  |  |  | ^I15_Ma_164^ | *^A. discapex^* | ^1^ | ^INMAC342-21^ | | ^-^ | | ^-^ | | ^Y+^ | |  |
|  |  |  |  |  |  |  |  |  |  | ^I15_Ma_165^ | *^A. discapex^* | ^1^ | ^INMAC343-21^ | | ^-^ | | ^-^ | | ^Y+^ | |  |
|  |  |  |  |  |  |  |  |  |  | ^I15_Ma_166^ | *^A. discapex^* | ^1^ | ^INMAC344-21^ | | ^-^ | | ^-^ | | ^Y+^ | |  |
|  |  |  |  |  |  |  |  |  |  | ^I15_Ma_167^ | *^A. discapex^* | ^1^ | ^INMAC345-21^ | | ^-^ | | ^-^ | | ^Y+^ | |  |
|  |  |  |  |  |  |  |  |  |  | ^I15_Ma_168^ | ^unidentified^ | ^1^ | ^INMAC346-21^ | | ^-^ | | ^-^ | | ^Ex^ | |  |
|  |  |  |  |  |  |  |  |  |  | ^-^ | *^A.^* ^juvenile^ | ^230^ | ^-^ | | ^-^ | | ^-^ | | ^-^ | |  |
|  |  |  | ^I15_62R_E_2^ | ^VF 1^ | ^23.88°S^ | ^69.62°E^ | ^3189.0^ | ^rock^ | ^claw^ | ^-^ | *^A.^* ^juvenile^ | ^11^ | ^-^ | | ^-^ | | ^-^ | | ^-^ | |  |
|  |  |  | ^I15_62R_B1_1^ | ^VF 1^ | ^23.88°S^ | ^69.62°E^ | ^3082.0^ | ^rock^ | ^claw^ | ^-^ | *^A. declivis^* | ^4^ | ^-^ | | ^-^ | | ^-^ | | ^-^ | |  |
|  |  |  |  |  |  |  |  |  |  | ^-^ | *^A. discapex^* | ^4^ | ^-^ | | ^-^ | | ^-^ | | ^-^ | |  |
|  |  |  | ^I15_62R_B1_2^ | ^VF 1^ | ^23.88°S^ | ^69.62°E^ | ^3082.0^ | ^rock^ | ^claw^ | ^-^ | *^A. declivis^* | ^5^ | ^-^ | | ^-^ | | ^-^ | | ^-^ | |  |
|  |  |  | ^I15_62R_B2_1^ | ^VF 1^ | ^23.88°S^ | ^69.62°E^ | ^3082.0^ | ^rock^ | ^claw^ | ^-^ | *^A. declivis^* | ^3^ | ^-^ | | ^-^ | | ^-^ | | ^-^ | |  |
|  |  |  | ^I15_62R_B2_2^ | ^VF 1^ | ^23.88°S^ | ^69.62°E^ | ^3082.0^ | ^rock^ | ^claw^ | ^-^ | *^A.^* ^juvenile^ | ^4^ | ^-^ | | ^-^ | | ^-^ | | ^-^ | |  |
|  |  |  | ^I15_62R_B3_2^ | ^VF 1^ | ^23.88°S^ | ^69.62°E^ | ^3082.0^ | ^rock^ | ^claw^ | ^I15_Ma_170^ | ^unidentified^ | ^1^ | ^INMAC446-21^ | | ^-^ | | ^-^ | | ^-^ | |  |
|  |  |  |  |  |  |  |  |  |  | ^-^ | *^A. declivis^* | ^2^ | ^-^ | | ^-^ | | ^-^ | | ^-^ | |  |
|  |  |  |  |  |  |  |  |  |  | ^-^ | ^unidentified^ | ^1^ | ^-^ | | ^-^ | | ^-^ | | ^-^ | |  |
| ^INDEX2018^ | ^21.11.2018^ | ^I18_59RO^ | ^I18_59RO_AFT_1^ | ^VF 4^ | ^27.65°S^ | ^73.88°E^ | ^2469.0^ | ^sediment, rock^ | ^scoop, claw^ | ^-^ | *^A. declivis^* | ^48^ | ^-^ | | ^-^ | | ^-^ | | ^-^ | |  |
|  |  |  |  |  |  |  |  |  |  | ^-^ | *^A. discapex^* | ^4^ | ^-^ | | ^-^ | | ^-^ | | ^-^ | |  |
|  |  |  | ^I18_59RO_AFT_2^ | ^VF 4^ | ^27.65°S^ | ^73.88°E^ | ^2469.0^ | ^sediment, rock^ | ^scoop, claw^ | ^-^ | *^A. declivis^* | ^11^ | ^-^ | | ^-^ | | ^-^ | | ^-^ | |  |
|  |  |  |  |  |  |  |  |  |  | ^-^ | *^A. discapex^* | ^5^ | ^-^ | | ^-^ | | ^-^ | | ^-^ | |  |
|  |  |  |  |  |  |  |  |  |  | ^-^ | *^A. laevapex^* | ^11^ | ^-^ | | ^-^ | | ^-^ | | ^-^ | |  |
|  | ^22.11.2018^ | ^I18_61RO^ | ^I18_61RO_4_1^ | ^VF 4^ | ^27.65°S^ | ^73.88°E^ | ^2482.0^ | ^rock^ | ^claw^ | ^-^ | *^A. declivis^* | ^8^ | ^-^ | | ^-^ | | ^-^ | | ^-^ | |  |
|  |  |  | ^I18_61RO_11_1^ | ^VF 4^ | ^27.65°S^ | ^73.88°E^ | ^2468.0^ | ^rock^ | ^claw^ | ^-^ | *^A. declivis^* | ^2^ | ^-^ | | ^-^ | | ^-^ | | ^-^ | |  |
|  |  |  |  |  |  |  |  |  |  | ^-^ | *^A. paucisculpta^* | ^1^ | ^-^ | | ^-^ | | ^-^ | | ^-^ | |  |
|  | ^23.11.2018^ | ^I18_63RO^ | ^I18_63RO_A_1^ | ^VF 4^ | ^27.65°S^ | ^73.88°E^ | ^2344.0^ | ^rock^ | ^claw^ | ^-^ | *^A. declivis^* | ^8^ | ^-^ | | ^-^ | | ^-^ | | ^-^ | |  |
|  |  |  | ^I18_63RO_A_2^ | ^VF 4^ | ^27.65°S^ | ^73.88°E^ | ^2344.0^ | ^rock^ | ^claw^ | ^-^ | *^A. discapex^* | ^8^ | ^-^ | | ^-^ | | ^-^ | | ^-^ | |  |
|  |  |  | ^I18_63RO_H_2^ | ^VF 4^ | ^27.65°S^ | ^73.88°E^ | ^2480.0^ | ^rock^ | ^claw^ | ^-^ | *^A. declivis^* | ^1^ | ^-^ | | ^-^ | | ^-^ | | ^-^ | |  |
|  |  |  | ^I18_63RO_J_1^ | ^VF 4^ | ^27.65°S^ | ^73.88°E^ | ^2480.0^ | ^rock^ | ^claw^ | ^-^ | *^A. discapex^* | ^6^ | ^-^ | | ^-^ | | ^-^ | | ^-^ | |  |
|  |  |  | ^I18_63RO_J_2^ | ^VF 4^ | ^27.65°S^ | ^73.88°E^ | ^2480.0^ | ^rock^ | ^claw^ | ^-^ | *^A. discapex^* | ^14^ | ^-^ | | ^-^ | | ^-^ | | ^-^ | |  |
|  |  |  | ^I18_63RO_SF_1^ | ^VF 4^ | ^27.65°S^ | ^73.88°E^ | ^2469.0^ | ^rock^ | ^claw^ | ^I18_Ma_256^ | *^A. declivis^* | ^1^ | ^INMAC278-21^ | | ^OM951062^ | | ^-^ | | ^Y^ | |  |
|  |  |  |  |  |  |  |  |  |  | ^I18_Ma_257^ | *^A. declivis^* | ^1^ | ^INMAC279-21^ | | ^OM951085^ | | ^-^ | | ^Y^ | |  |
|  |  |  |  |  |  |  |  |  |  | ^I18_Ma_258^ | *^A. declivis^* | ^1^ | ^INMAC280-21^ | | ^OM951064^ | | ^-^ | | ^Y^ | |  |
|  |  |  |  |  |  |  |  |  |  | ^I18_Ma_259^ | *^A. declivis^* | ^1^ | ^INMAC281-21^ | | ^OM951124^ | | ^-^ | | ^Y^ | |  |
|  |  |  |  |  |  |  |  |  |  | ^I18_Ma_260^ | *^A. declivis^* | ^1^ | ^INMAC282-21^ | | ^OM951029^ | | ^-^ | | ^Y^ | |  |
|  |  |  |  |  |  |  |  |  |  | ^I18_Ma_261^ | *^A. declivis^* | ^1^ | ^INMAC283-21^ | | ^OM951023^ | | ^-^ | | ^Y^ | |  |
|  |  |  |  |  |  |  |  |  |  | ^I18_Ma_262^ | *^A. declivis^* | ^1^ | ^INMAC284-21^ | | ^OM951109^ | | ^-^ | | ^Y^ | |  |
|  |  |  |  |  |  |  |  |  |  | ^I18_Ma_263^ | *^A. declivis^* | ^1^ | ^INMAC285-21^ | | ^OM951077^ | | ^-^ | | ^Y^ | |  |
|  |  |  |  |  |  |  |  |  |  | ^I18_Ma_264^ | *^A. declivis^* | ^1^ | ^INMAC286-21^ | | ^OM951043^ | | ^-^ | | ^Y^ | |  |
|  |  |  |  |  |  |  |  |  |  | ^I18_Ma_265^ | *^A. declivis^* | ^1^ | ^INMAC287-21^ | | ^-^ | | ^-^ | | ^Y^ | |  |
|  |  |  |  |  |  |  |  |  |  | ^I18_Ma_266^ | *^A. declivis^* | ^1^ | ^INMAC288-21^ | | ^OM951112^ | | ^-^ | | ^Y^ | |  |
|  |  |  |  |  |  |  |  |  |  | ^I18_Ma_267^ | *^A. declivis^* | ^1^ | ^INMAC289-21^ | | ^OM951099^ | | ^-^ | | ^Y^ | |  |
|  |  |  |  |  |  |  |  |  |  | ^I18_Ma_268^ | *^A. declivis^* | ^1^ | ^INMAC290-21^ | | ^OM951061^ | | ^-^ | | ^Y^ | |  |
|  |  |  |  |  |  |  |  |  |  | ^I18_Ma_269^ | *^A. declivis^* | ^1^ | ^INMAC291-21^ | | ^OM951128^ | | ^-^ | | ^Y^ | |  |
|  |  |  |  |  |  |  |  |  |  | ^I18_Ma_270^ | *^A. declivis^* | ^1^ | ^INMAC292-21^ | | ^-^ | | ^-^ | | ^Y+^ | |  |
|  |  |  |  |  |  |  |  |  |  | ^I18_Ma_271^ | *^A. paucisculpta^* | ^1^ | ^INMAC293-21^ | | ^OM951132^ | | ^-^ | | ^Y^ | |  |
|  |  |  |  |  |  |  |  |  |  | ^I18_Ma_272^ | *^A. paucisculpta^* | ^1^ | ^INMAC294-21^ | | ^OM951093^ | | ^-^ | | ^Y^ | |  |
|  |  |  |  |  |  |  |  |  |  | ^I18_Ma_273^ | *^A. declivis^* | ^1^ | ^INMAC295-21^ | | ^OM951076^ | | ^-^ | | ^Y^ | |  |
|  |  |  |  |  |  |  |  |  |  | ^I18_Ma_274^ | *^A. declivis^* | ^1^ | ^INMAC296-21^ | | ^OM951078^ | | ^-^ | | ^Y^ | |  |
|  |  |  |  |  |  |  |  |  |  | ^I18_Ma_275^ | *^A. declivis^* | ^1^ | ^INMAC297-21^ | | ^-^ | | ^-^ | | ^Y^ | |  |
|  |  |  |  |  |  |  |  |  |  | ^I18_Ma_276^ | *^A. declivis^* | ^1^ | ^INMAC298-21^ | | ^OM951118^ | | ^-^ | | ^Y^ | |  |
|  |  |  |  |  |  |  |  |  |  | ^I18_Ma_277^ | *^A. declivis^* | ^1^ | ^INMAC299-21^ | | ^OM951075^ | | ^-^ | | ^Y^ | |  |
|  |  |  |  |  |  |  |  |  |  | ^I18_Ma_278^ | *^A. declivis^* | ^1^ | ^INMAC300-21^ | | ^OM951108^ | | ^-^ | | ^Y^ | |  |
|  |  |  |  |  |  |  |  |  |  | ^I18_Ma_279^ | *^A. declivis^* | ^1^ | ^INMAC301-21^ | | ^OM951032^ | | ^-^ | | ^Y^ | |  |
|  |  |  |  |  |  |  |  |  |  | ^-^ | *^A. declivis^* | ^23^ | ^-^ | | ^-^ | | ^-^ | | ^-^ | |  |
|  |  |  | ^I18_63RO_AFT_1^ | ^VF 4^ | ^27.65°S^ | ^73.88°E^ | ^2469.0^ | ^rock^ | ^claw^ | ^-^ | *^A. declivis^* | ^24^ | ^-^ | | ^-^ | | ^-^ | | ^-^ | |  |
|  |  |  |  |  |  |  |  |  |  | ^-^ | *^A. paucisculpta^* | ^3^ | ^-^ | | ^-^ | | ^-^ | | ^-^ | |  |
| ^INDEX2019^ | ^11.11.2019^ | ^I19_031RO^ | ^I19_031RO_SF_01^ | ^VF 1^ | ^23.88°S^ | ^69.62°E^ | ^3082.0^ | ^rock^ | ^claw^ | ^I19_Ma_86^ | *^A. discapex^* | ^1^ | ^INMAC430-21^ | | ^-^ | | ^SMF 358979^ | | ^-^ | |  |
|  |  |  |  |  |  |  |  |  |  | ^-^ | *^A. discapex^* | ^1^ | ^-^ | | ^-^ | | ^SMF 358980^ | | ^-^ | |  |
|  |  |  |  |  |  |  |  |  |  | ^I19_Ma_92^ | *^A. declivis^* | ^1^ | ^INMAC436-21^ | | ^OM951021^ | | ^SMF 358985^ | | ^-^ | |  |
|  |  |  |  |  |  |  |  |  |  | ^I19_Ma_93^ | *^A. declivis^* | ^1^ | ^INMAC437-21^ | | ^-^ | | ^SMF 358986^ | | ^-^ | |  |
|  |  |  |  |  |  |  |  |  |  | ^-^ | *^A. declivis^* | ^1^ | ^-^ | | ^-^ | | ^-^ | | ^-^ | |  |
|  |  |  |  |  |  |  |  |  |  | ^-^ | *^A. discapex^* | ^5^ | ^-^ | | ^-^ | | ^-^ | | ^-^ | |  |
|  |  |  | ^I19_031RO_SF_02^ | ^VF 1^ | ^23.88°S^ | ^69.62°E^ | ^3082.0^ | ^rock^ | ^claw^ | ^I19_Ma_78^ | *^A. declivis^* | ^1^ | ^INMAC422-21^ | | ^-^ | | ^-^ | | ^-^ | |  |
|  |  |  |  |  |  |  |  |  |  | ^I19_Ma_79^ | *^A. discapex^* | ^1^ | ^INMAC423-21^ | | ^-^ | | ^SMF 358983^ | | ^-^ | |  |
|  |  |  |  |  |  |  |  |  |  | ^I19_Ma_80^ | *^A. discapex^* | ^1^ | ^INMAC424-21^ | | ^-^ | | ^SMF 358981^ | | ^-^ | |  |
|  |  |  |  |  |  |  |  |  |  | ^I19_Ma_81^ | *^A. discapex^* | ^1^ | ^INMAC425-21^ | | ^-^ | | ^SMF 358982^ | | ^-^ | |  |
|  | ^12.11.2019^ | ^I19_033RO^ | ^I19_033RO_A_01^ | ^Gauss^ | ^23.87°S^ | ^69.62°E^ | ^3031.7^ | ^rock^ | ^claw^ | ^-^ | *^A. discapex^* | ^3^ | ^-^ | | ^-^ | | ^-^ | | ^-^ | |  |
|  |  |  | ^I19_033RO_B_01^ | ^Gauss^ | ^23.87°S^ | ^69.62°E^ | ^3032.3^ | ^rock^ | ^claw^ | ^-^ | *^A. discapex^* | ^1^ | ^-^ | | ^-^ | | ^-^ | | ^-^ | |  |
|  |  |  | ^I19_033RO_SF_01^ | ^Gauss^ | ^23.87°S^ | ^69.62°E^ | ^2980.0^ | ^rock^ | ^claw^ | ^I19_Ma_3^ | *^A. declivis^* | ^1^ | ^INMAC347-21^ | | ^OM951073^ | | ^-^ | | ^Y^ | |  |
|  |  |  |  |  |  |  |  |  |  | ^I19_Ma_4^ | *^A. declivis^* | ^1^ | ^INMAC348-21^ | | ^OM951070^ | | ^-^ | | ^Y^ | |  |
|  |  |  |  |  |  |  |  |  |  | ^I19_Ma_5^ | *^A. discapex^* | ^1^ | ^INMAC349-21^ | | ^OM951074^ | | ^-^ | | ^Y^ | |  |
|  |  |  |  |  |  |  |  |  |  | ^I19_Ma_6^ | *^A. declivis^* | ^1^ | ^INMAC350-21^ | | ^OM951028^ | | ^-^ | | ^Y^ | |  |
|  |  |  |  |  |  |  |  |  |  | ^I19_Ma_7^ | *^A. declivis^* | ^1^ | ^INMAC351-21^ | | ^OM951094^ | | ^-^ | | ^Y^ | |  |
|  |  |  | ^I19_033RO_Pasa_01^ | ^Gauss^ | ^23.87°S^ | ^69.62°E^ | ^2980.0^ | ^rock^ | ^claw^ | ^I19_Ma_8^ | *^A. laevapex^* | ^1^ | ^INMAC352-21^ | | ^OM951098^ | | ^-^ | | ^Y^ | |  |
|  |  |  |  |  |  |  |  |  |  | ^I19_Ma_9^ | *^A. laevapex^* | ^1^ | ^INMAC353-21^ | | ^OM951130^ | | ^-^ | | ^Y^ | |  |
|  |  |  |  |  |  |  |  |  |  | ^I19_Ma_10^ | *^A. laevapex^* | ^1^ | ^INMAC354-21^ | | ^OM951071^ | | ^-^ | | ^Y^ | |  |
|  |  |  |  |  |  |  |  |  |  | ^I19_Ma_11^ | *^A. laevapex^* | ^1^ | ^INMAC355-21^ | | ^OM951057^ | | ^-^ | | ^Y^ | |  |
|  |  |  |  |  |  |  |  |  |  | ^I19_Ma_12^ | *^A. laevapex^* | ^1^ | ^INMAC356-21^ | | ^OM951033^ | | ^-^ | | ^Y^ | |  |
|  |  |  |  |  |  |  |  |  |  | ^I19_Ma_13^ | *^A. laevapex^* | ^1^ | ^INMAC357-21^ | | ^OM951037^ | | ^-^ | | ^Y^ | |  |
|  |  |  |  |  |  |  |  |  |  | ^I19_Ma_14^ | *^A. laevapex^* | ^1^ | ^INMAC358-21^ | | ^OM951125^ | | ^-^ | | ^Y^ | |  |
|  |  |  |  |  |  |  |  |  |  | ^I19_Ma_15^ | *^A. laevapex^* | ^1^ | ^INMAC359-21^ | | ^OM951091^ | | ^-^ | | ^Y^ | |  |
|  |  |  |  |  |  |  |  |  |  | ^I19_Ma_16^ | *^A. laevapex^* | ^1^ | ^INMAC360-21^ | | ^OM951101^ | | ^-^ | | ^Y^ | |  |
|  |  |  |  |  |  |  |  |  |  | ^I19_Ma_17^ | *^A. laevapex^* | ^1^ | ^INMAC361-21^ | | ^OM951119^ | | ^-^ | | ^Y^ | |  |
|  |  |  |  |  |  |  |  |  |  | ^I19_Ma_18^ | *^A. laevapex^* | ^1^ | ^INMAC362-21^ | | ^OM951096^ | | ^-^ | | ^Ex^ | |  |
|  |  |  |  |  |  |  |  |  |  | ^I19_Ma_19^ | *^A. laevapex^* | ^1^ | ^INMAC363-21^ | | ^OM951069^ | | ^-^ | | ^Y^ | |  |
|  |  |  |  |  |  |  |  |  |  | ^I19_Ma_20^ | *^A. laevapex^* | ^1^ | ^INMAC364-21^ | | ^OM951060^ | | ^-^ | | ^Y^ | |  |
|  |  |  |  |  |  |  |  |  |  | ^I19_Ma_21^ | *^A. laevapex^* | ^1^ | ^INMAC365-21^ | | ^-^ | | ^-^ | | ^Y^ | |  |
|  |  |  |  |  |  |  |  |  |  | ^I19_Ma_22^ | *^A. laevapex^* | ^1^ | ^INMAC366-21^ | | ^OM951042^ | | ^-^ | | ^Y^ | |  |
|  |  |  |  |  |  |  |  |  |  | ^I19_Ma_23^ | *^A. laevapex^* | ^1^ | ^INMAC367-21^ | | ^OM951022^ | | ^-^ | | ^Y^ | |  |
|  |  |  |  |  |  |  |  |  |  | ^I19_Ma_24^ | *^A. laevapex^* | ^1^ | ^INMAC368-21^ | | ^OM951113^ | | ^-^ | | ^Y^ | |  |
|  |  |  |  |  |  |  |  |  |  | ^I19_Ma_25^ | *^A. laevapex^* | ^1^ | ^INMAC369-21^ | | ^OM951121^ | | ^-^ | | ^Y^ | |  |
|  |  |  |  |  |  |  |  |  |  | ^I19_Ma_26^ | *^A. laevapex^* | ^1^ | ^INMAC370-21^ | | ^OM951053^ | | ^-^ | | ^Y^ | |  |
|  |  |  |  |  |  |  |  |  |  | ^I19_Ma_94^ | *^A. declivis^* | ^1^ | ^INMAC438-21^ | | ^-^ | | ^SMF 358984^ | | ^-^ | |  |
|  |  |  |  |  |  |  |  |  |  | ^I19_Ma_95^ | *^A. laevapex^* | ^1^ | ^INMAC439-21^ | | ^-^ | | ^-^ | | ^-^ | |  |
|  |  |  |  |  |  |  |  |  |  | ^I19_Ma_96^ | *^A. laevapex^* | ^1^ | ^INMAC440-21^ | | ^OM951086^ | | ^-^ | | ^-^ | |  |
|  |  |  |  |  |  |  |  |  |  | ^I19_Ma_97^ | *^A. laevapex^* | ^1^ | ^INMAC441-21^ | | ^OM951117^ | | ^-^ | | ^-^ | |  |
|  |  |  |  |  |  |  |  |  |  | ^I19_Ma_98^ | *^A. laevapex^* | ^1^ | ^INMAC442-21^ | | ^OM951055^ | | ^-^ | | ^-^ | |  |
|  |  |  |  |  |  |  |  |  |  | ^I19_Ma_99^ | *^A. laevapex^* | ^1^ | ^INMAC443-21^ | | ^OM951041^ | | ^-^ | | ^-^ | |  |
|  |  |  |  |  |  |  |  |  |  | ^I19_Ma_100^ | ^unidentified^ | ^1^ | ^INMAC444-21^ | | ^-^ | | ^-^ | | ^-^ | |  |
|  | ^14.11.2019^ | ^I19_036RO^ | ^I19_036RO_C_01^ | ^VF 3^ | ^25.47°S^ | ^69.93°E^ | ^2754.2^ | ^rock^ | ^claw^ | ^-^ | *^A. declivis^* | ^1^ | ^-^ | | ^-^ | | ^-^ | | ^-^ | |  |
|  |  |  | ^I19_036RO_M_01^ | ^VF 3^ | ^25.47°S^ | ^69.93°E^ | ^2634.2^ | ^rock^ | ^claw^ | ^-^ | *^A. declivis^* | ^3^ | ^-^ | | ^-^ | | ^-^ | | ^-^ | |  |
|  |  |  | ^I19_036RO_Pasa_01^ | ^VF 3^ | ^25.47°S^ | ^69.93°E^ | ^2628.0^ | ^rock^ | ^claw^ | ^-^ | *^A. declivis^* | ^1^ | ^-^ | | ^-^ | | ^-^ | | ^-^ | |  |
|  | ^16.11.2019^ | ^I19_042RO^ | ^I19_042RO_B_01^ | ^VF 3^ | ^25.47°S^ | ^69.93°E^ | ^2932.5^ | ^rock^ | ^claw^ | ^-^ | *^A. discapex^* | ^1^ | ^-^ | | ^-^ | | ^-^ | | ^-^ | |  |
|  |  |  | ^I19_042RO_B_02^ | ^VF 3^ | ^25.47°S^ | ^69.93°E^ | ^2932.5^ | ^rock^ | ^claw^ | ^-^ | *^A. discapex^* | ^5^ | ^-^ | | ^-^ | | ^-^ | | ^-^ | |  |
|  |  |  | ^I19_042RO_D_01^ | ^VF 3^ | ^25.47°S^ | ^69.93°E^ | ^2934.6^ | ^rock^ | ^claw^ | ^-^ | *^A. declivis^* | ^1^ | ^-^ | | ^-^ | | ^-^ | | ^-^ | |  |
|  |  |  | ^I19_042RO_PF_01^ | ^VF 3^ | ^25.47°S^ | ^69.93°E^ | ^2628.0^ | ^rock^ | ^claw^ | ^I19_Ma_27^ | *^A. discapex^* | ^1^ | ^INMAC371-21^ | | ^OM951102^ | | ^-^ | | ^Y^ | |  |
|  |  |  |  |  |  |  |  |  |  | ^I19_Ma_28^ | *^A. discapex^* | ^1^ | ^INMAC372-21^ | | ^OM951066^ | | ^-^ | | ^Y^ | |  |
|  |  |  |  |  |  |  |  |  |  | ^I19_Ma_29^ | *^A. discapex^* | ^1^ | ^INMAC373-21^ | | ^OM951046^ | | ^-^ | | ^Y^ | |  |
|  |  |  |  |  |  |  |  |  |  | ^I19_Ma_30^ | *^A. discapex^* | ^1^ | ^INMAC374-21^ | | ^OM951038^ | | ^-^ | | ^Y^ | |  |
|  |  |  |  |  |  |  |  |  |  | ^I19_Ma_31^ | *^A. discapex^* | ^1^ | ^INMAC375-21^ | | ^OM951054^ | | ^-^ | | ^Y^ | |  |
|  |  |  |  |  |  |  |  |  |  | ^I19_Ma_32^ | *^A. discapex^* | ^1^ | ^INMAC376-21^ | | ^OM951126^ | | ^-^ | | ^Y^ | |  |
|  |  |  |  |  |  |  |  |  |  | ^I19_Ma_33^ | *^A. discapex^* | ^1^ | ^INMAC377-21^ | | ^OM951080^ | | ^-^ | | ^Y^ | |  |
|  |  |  |  |  |  |  |  |  |  | ^I19_Ma_34^ | *^A. discapex^* | ^1^ | ^INMAC378-21^ | | ^OM951047^ | | ^-^ | | ^Y^ | |  |
|  |  |  |  |  |  |  |  |  |  | ^I19_Ma_35^ | *^A. discapex^* | ^1^ | ^INMAC379-21^ | | ^OM951050^ | | ^-^ | | ^Ex^ | |  |
|  |  |  |  |  |  |  |  |  |  | ^I19_Ma_36^ | *^A. discapex^* | ^1^ | ^INMAC380-21^ | | ^OM951100^ | | ^-^ | | ^Y^ | |  |
|  |  |  |  |  |  |  |  |  |  | ^I19_Ma_37^ | ^unidentified^ | ^1^ | ^INMAC381-21^ | | ^-^ | | ^-^ | | ^Ex^ | |  |
|  |  |  |  |  |  |  |  |  |  | ^I19_Ma_38^ | *^A. discapex^* | ^1^ | ^INMAC382-21^ | | ^OM951079^ | | ^-^ | | ^Y^ | |  |
|  |  |  |  |  |  |  |  |  |  | ^I19_Ma_39^ | *^A. discapex^* | ^1^ | ^INMAC383-21^ | | ^OM951090^ | | ^-^ | | ^Y^ | |  |
|  |  |  |  |  |  |  |  |  |  | ^I19_Ma_40^ | *^A. discapex^* | ^1^ | ^INMAC384-21^ | | ^OM951030^ | | ^-^ | | ^Y^ | |  |
|  |  |  |  |  |  |  |  |  |  | ^I19_Ma_41^ | *^A. discapex^* | ^1^ | ^INMAC385-21^ | | ^OM951087^ | | ^-^ | | ^Y^ | |  |
|  |  |  |  |  |  |  |  |  |  | ^I19_Ma_42^ | *^A. discapex^* | ^1^ | ^INMAC386-21^ | | ^OM951131^ | | ^-^ | | ^Y^ | |  |
|  |  |  |  |  |  |  |  |  |  | ^I19_Ma_43^ | *^A. discapex^* | ^1^ | ^INMAC387-21^ | | ^OM951129^ | | ^-^ | | ^Ex^ | |  |
|  |  |  |  |  |  |  |  |  |  | ^I19_Ma_44^ | *^A. discapex^* | ^1^ | ^INMAC388-21^ | | ^OM951059^ | | ^-^ | | ^Ex^ | |  |
|  |  |  |  |  |  |  |  |  |  | ^I19_Ma_45^ | *^A. discapex^* | ^1^ | ^INMAC389-21^ | | ^OM951110^ | | ^-^ | | ^Y^ | |  |
|  |  |  |  |  |  |  |  |  |  | ^I19_Ma_46^ | *^A. discapex^* | ^1^ | ^INMAC390-21^ | | ^-^ | | ^-^ | | ^Y+^ | |  |
|  |  |  |  |  |  |  |  |  |  | ^I19_Ma_47^ | *^A. discapex^* | ^1^ | ^INMAC391-21^ | | ^OM951056^ | | ^-^ | | ^Y^ | |  |
|  |  |  |  |  |  |  |  |  |  | ^I19_Ma_48^ | *^A. discapex^* | ^1^ | ^INMAC392-21^ | | ^OM951027^ | | ^-^ | | ^Y^ | |  |
|  |  |  |  |  |  |  |  |  |  | ^I19_Ma_49^ | *^A. discapex^* | ^1^ | ^INMAC393-21^ | | ^OM951084^ | | ^-^ | | ^Y^ | |  |
|  |  |  |  |  |  |  |  |  |  | ^I19_Ma_50^ | *^A. discapex^* | ^1^ | ^INMAC394-21^ | | ^OM951068^ | | ^-^ | | ^Y^ | |  |
|  |  |  |  |  |  |  |  |  |  | ^I19_Ma_82^ | *^A. discapex^* | ^1^ | ^INMAC426-21^ | | ^-^ | | ^-^ | | ^-^ | |  |
|  |  |  |  |  |  |  |  |  |  | ^I19_Ma_83^ | *^A. discapex^* | ^1^ | ^INMAC427-21^ | | ^-^ | | ^-^ | | ^-^ | |  |
|  |  |  |  |  |  |  |  |  |  | ^I19_Ma_84^ | *^A. discapex^* | ^1^ | ^INMAC428-21^ | | ^-^ | | ^SMF 358976^ | | ^-^ | |  |
|  |  |  |  |  |  |  |  |  |  | ^I19_Ma_85^ | *^A. discapex^* | ^1^ | ^INMAC429-21^ | | ^-^ | | ^SMF 358978^ | | ^-^ | |  |
|  |  |  |  |  |  |  |  |  |  | ^-^ | *^A. discapex^* | ^1^ | ^-^ | | ^-^ | | ^SMF 358977^ | | ^-^ | |  |
|  |  |  | ^I19_042RO_SF_01^ | ^VF 3^ | ^25.47°S^ | ^69.93°E^ | ^2628.0^ | ^rock^ | ^claw^ | ^I19_Ma_89^ | *^A. declivis^* | ^1^ | ^INMAC433-21^ | | ^-^ | | ^SMF 358987^ | | ^-^ | |  |
|  |  |  |  |  |  |  |  |  |  | ^I19_Ma_90^ | *^A.^* ^sp. 2 DZMB_2021_0096^ | ^1^ | ^INMAC434-21^ | | ^OM951107^ | | ^-^ | | ^-^ | |  |
|  | ^05.12.2019^ | ^I19_102RO^ | ^I19_102RO_G_02^ | ^VF 5^ | ^27.63°S^ | ^73.87°E^ | ^2546.9^ | ^rock^ | ^claw^ | ^I19_Ma_87^ | *^A. declivis^* | ^1^ | ^INMAC431-21^ | | ^-^ | | ^SMF 358988^ | | ^-^ | |  |
|  |  |  |  |  |  |  |  |  |  | ^I19_Ma_88^ | *^A. declivis^* | ^1^ | ^INMAC432-21^ | | ^OM951052^ | | ^-^ | | ^-^ | |  |
|  |  |  | ^I19_102RO_K_01^ | ^VF 5^ | ^27.63°S^ | ^73.87°E^ | ^2576.7^ | ^rock^ | ^claw^ | ^I19_Ma_51^ | *^A. declivis^* | ^1^ | ^INMAC395-21^ | | ^OM951106^ | | ^-^ | | ^Y^ | |  |
|  |  |  | ^I19_102RO_SF_02^ | ^VF 5^ | ^27.63°S^ | ^73.87°E^ | ^2532.0^ | ^rock^ | ^claw^ | **^-^** | *^A. laevapex^* | ^4^ | **^-^** | | **^-^** | | ^-^ | | ^-^ | |  |
|  |  |  | ^I19_102RO_PASA_01^ | ^VF 5^ | ^27.63°S^ | ^73.87°E^ | ^2532.0^ | ^rock^ | ^claw^ | ^I19_Ma_54^ | *^A. declivis^* | ^1^ | ^INMAC398-21^ | | ^OM951067^ | | ^-^ | | ^Y^ | |  |
|  |  |  |  |  |  |  |  |  |  | ^I19_Ma_55^ | *^A. declivis^* | ^1^ | ^INMAC399-21^ | | ^OM951039^ | | ^-^ | | ^Y^ | |  |
|  |  |  |  |  |  |  |  |  |  | ^I19_Ma_56^ | *^A. declivis^* | ^1^ | ^INMAC400-21^ | | ^OM951092^ | | ^-^ | | ^Y^ | |  |
|  |  |  |  |  |  |  |  |  |  | ^I19_Ma_57^ | *^A. declivis^* | ^1^ | ^INMAC401-21^ | | ^OM951048^ | | ^-^ | | ^Y^ | |  |
|  |  |  |  |  |  |  |  |  |  | ^I19_Ma_58^ | *^A. declivis^* | ^1^ | ^INMAC402-21^ | | ^OM951083^ | | ^-^ | | ^Ex^ | |  |
|  |  |  |  |  |  |  |  |  |  | ^I19_Ma_59^ | *^A. declivis^* | ^1^ | ^INMAC403-21^ | | ^OM951081^ | | ^-^ | | ^Y^ | |  |
|  |  |  |  |  |  |  |  |  |  | ^I19_Ma_60^ | *^A. declivis^* | ^1^ | ^INMAC404-21^ | | ^OM951026^ | | ^-^ | | ^Y^ | |  |
|  |  |  |  |  |  |  |  |  |  | ^I19_Ma_61^ | *^A. declivis^* | ^1^ | ^INMAC405-21^ | | ^OM951072^ | | ^-^ | | ^Y^ | |  |
|  |  |  |  |  |  |  |  |  |  | ^I19_Ma_62^ | *^A. laevapex^* | ^1^ | ^INMAC406-21^ | | ^OM951116^ | | ^-^ | | ^Y^ | |  |
|  |  |  |  |  |  |  |  |  |  | ^I19_Ma_63^ | *^A. declivis^* | ^1^ | ^INMAC407-21^ | | ^OM951040^ | | ^-^ | | ^Y^ | |  |
|  |  |  |  |  |  |  |  |  |  | ^I19_Ma_64^ | *^A. declivis^* | ^1^ | ^INMAC408-21^ | | ^OM951031^ | | ^-^ | | ^Y^ | |  |
|  |  |  |  |  |  |  |  |  |  | ^I19_Ma_65^ | *^A. laevapex^* | ^1^ | ^INMAC409-21^ | | ^OM951049^ | | ^-^ | | ^Y^ | |  |
|  |  |  |  |  |  |  |  |  |  | ^I19_Ma_66^ | *^A. declivis^* | ^1^ | ^INMAC410-21^ | | ^-^ | | ^-^ | | ^Ex^ | |  |
|  |  |  |  |  |  |  |  |  |  | ^I19_Ma_67^ | *^A. declivis^* | ^1^ | ^INMAC411-21^ | | ^OM951044^ | | ^-^ | | ^Y^ | |  |
|  |  |  |  |  |  |  |  |  |  | ^I19_Ma_68^ | *^A. declivis^* | ^1^ | ^INMAC412-21^ | | ^OM951045^ | | ^-^ | | ^Ex^ | |  |
|  |  |  |  |  |  |  |  |  |  | ^I19_Ma_69^ | *^A. laevapex^* | ^1^ | ^INMAC413-21^ | | ^OM951088^ | | ^-^ | | ^Y^ | |  |
|  |  |  |  |  |  |  |  |  |  | ^I19_Ma_70^ | *^A. laevapex^* | ^1^ | ^INMAC414-21­^ | | ^OM951120^ | | ^-^ | | ^Y^ | |  |
|  | ^06.12.2019^ | ^I19_104RO^ | ^I19_104RO_PC2_02^ | ^VF 5^ | ^27.63°S^ | ^73.87°E^ | ^2617.8^ | ^sediment^ | ^push corer^ | ^I19_Ma_91^ | *^A. paucisculpta^* | ^1^ | ^INMAC435-21^ | | ^-^ | | ^SMF 358993^ | | ^-^ | |  |
|  |  |  |  |  |  |  |  |  |  | ^-^ | ^unidentified^ | ^1^ | ^-^ | | ^-^ | | ^-^ | | ^-^ | |  |
|  | ^07.12.2019^ | ^I19_106RO^ | ^I19_106RO_E_01^ | ^VF 5^ | ^27.63°S^ | ^73.87°E^ | ^2620.9^ | ^rock^ | ^claw^ | ^I19_Ma_52^ | *^A. paucisculpta^* | ^1^ | ^INMAC396-21^ | | ^OM951115^ | | ^-^ | | ^Ex^ | |  |
|  |  |  |  |  |  |  |  |  |  | ^I19_Ma_53^ | *^A.^* ^sp. 1 DZMB_2021_0095^ | ^1^ | ^INMAC397-21^ | | ^OM951065^ | | ^-^ | | ^Y^ | |  |
|  |  |  | ^I19_106RO_SF_01^ | ^VF 5^ | ^27.63°S^ | ^73.87°E^ | ^2532.0^ | ^sediment, rock^ | ^claw, slurp gun^ | ^I19_Ma_71^ | *^A. paucisculpta^* | ^1^ | ^INMAC415-21^ | | ^OM951133^ | | ^-^ | | ^Y^ | |  |
|  | ^11.12.2019^ | ^I19_127RO^ | ^I19_127RO_F_01^ | ^VF 4^ | ^27.65°S^ | ^73.88°E^ | ^2457.2^ | ^rock^ | ^claw^ | ^I19_Ma_77^ | *^A. declivis^* | ^1^ | ^INMAC421-21^ | | ^-^ | | ^-^ | | ^-^ | |  |
|  |  |  | ^I19_127RO_F_02^ | ^VF 4^ | ^27.65°S^ | ^73.88°E^ | ^2457.2^ | ^rock^ | ^claw^ | ^-^ | *^A. laevapex^* | ^8^ | ^-^ | | ^-^ | | ^-^ | | ^-^ | |  |
|  |  |  | ^I19_127RO_H_01^ | ^VF 4^ | ^27.65°S^ | ^73.88°E^ | ^2471.1^ | ^rock^ | ^claw^ | ^I19_Ma_72^ | *^A. laevapex^* | ^1^ | ^INMAC416-21^ | | ^-^ | | ^-^ | | ^-^ | |  |
|  |  |  | ^I19_127RO_H_02^ | ^VF 4^ | ^27.65°S^ | ^73.88°E^ | ^2471.1^ | ^rock^ | ^claw^ | ^-^ | *^A. laevapex^* | ^10^ | ^-^ | | ^-^ | | ^-^ | | ^-^ | |  |
|  |  |  | ^I19_127RO_SF_01^ | ^VF 4^ | ^27.65°S^ | ^73.88°E^ | ^2469.0^ | ^rock^ | ^claw^ | ^-^ | *^A. laevapex^* | ^16^ | ^-^ | | ^-^ | | ^-^ | | ^-^ | |  |
|  |  |  | ^I19_127RO_SF_02^ | ^VF 4^ | ^27.65°S^ | ^73.88°E^ | ^2469.0^ | ^rock^ | ^claw^ | ^I19_Ma_73^ | *^A. laevapex^* | ^1^ | ^INMAC417-21^ | | ^-^ | | ^-^ | | ^-^ | |  |
|  |  |  |  |  |  |  |  |  |  | ^I19_Ma_74^ | *^A. laevapex^* | ^1^ | ^INMAC418-21^ | | ^-^ | | ^SMF 358989^ | | ^-^ | |  |
|  |  |  |  |  |  |  |  |  |  | ^I19_Ma_75^ | *^A. laevapex^* | ^1^ | ^INMAC419-21^ | | ^-^ | | ^SMF 358990^ | | ^-^ | |  |
|  |  |  |  |  |  |  |  |  |  | ^I19_Ma_76^ | *^A. laevapex^* | ^1^ | ^INMAC420-21^ | | ^-^ | | ^SMF 358991^ | | ^-^ | |  |
|  |  |  |  |  |  |  |  |  |  | ^-^ | *^A. laevapex^* | ^9^ | ^-^ | | ^-^ | | ^-^ | | ^-^ | |  |
|  |  |  | ^I19_127RO_PF_02^ | ^VF 4^ | ^27.65°S^ | ^73.88°E^ | ^2469.0^ | ^rock^ | ^claw^ | ^-^ | *^A. laevapex^* | ^8^ | ^-^ | | ^-^ | | ^-^ | | ^-^ | |  |
|  |  |  | ^I19_127RO_SG2_02^ | ^VF 4^ | ^27.65°S^ | ^73.88°E^ | ^2469.5^ | ^sediment^ | ^slurp gun^ | ^-^ | *^A. laevapex^* | ^1^ | ^-^ | | ^-^ | | ^-^ | | ^-^ | |  |

Table S2: Used adapters and primers for the 2b-RAD analysis. The asterisks indicate the oligo pairs that require hybridization.

| **Primer Type** | **Primer Name** | **Sequence (5' to 3' direction)** |
| --- | --- | --- |
| Index Primer | ILLP7_1 | CAAGCAGAAGACGGCATACGAGATATTACTCGGTGACTGGAGTTCAGACGTGTGCTCTTCCGAT |
|  | ILLP7_2 | CAAGCAGAAGACGGCATACGAGATTCCGGAGAGTGACTGGAGTTCAGACGTGTGCTCTTCCGAT |
|  | ILLP7_3 | CAAGCAGAAGACGGCATACGAGATCGCTCATTGTGACTGGAGTTCAGACGTGTGCTCTTCCGAT |
|  | ILLP7_4 | CAAGCAGAAGACGGCATACGAGATGAGATTCCGTGACTGGAGTTCAGACGTGTGCTCTTCCGAT |
|  | ILLP7_5 | CAAGCAGAAGACGGCATACGAGATATTCAGAAGTGACTGGAGTTCAGACGTGTGCTCTTCCGAT |
|  | ILLP5_1 | AATGATACGGCGACCACCGAGATCTACACTGAACCTTACACTCTTTCCCTACACGACGCTCTTCCGATCT |
|  | ILLP5_2 | AATGATACGGCGACCACCGAGATCTACACTAAGACACACACTCTTTCCCTACACGACGCTCTTCCGATCT |
|  | ILLP5_3 | AATGATACGGCGACCACCGAGATCTACACGGCTACTTACACTCTTTCCCTACACGACGCTCTTCCGATCT |
|  | ILLP5_4 | AATGATACGGCGACCACCGAGATCTACACCTAGAACAACACTCTTTCCCTACACGACGCTCTTCCGATCT |
|  | ILLP5_5 | AATGATACGGCGACCACCGAGATCTACACAAGACCTAACACTCTTTCCCTACACGACGCTCTTCCGATCT |
| Adapter F (nonspecific)* | 5ILL-NG | CTACACGACGCTCTTCCGATCTNNRWCCNG |
|  | antiIll-NNRW | GGWYNNAGATCGG |
| Adapter R (specific)* | 3illBC1 | CAGACGTGTGCTCTTCCGATCTACACNG |
|  | 3illBC2 | CAGACGTGTGCTCTTCCGATCTGTCTNG |
|  | 3illBC3 | CAGACGTGTGCTCTTCCGATCTTGGTNG |
|  | 3illBC4 | CAGACGTGTGCTCTTCCGATCTCACTNG |
|  | 3illBC5 | CAGACGTGTGCTCTTCCGATCTGATGNG |
|  | antiBC1 | GTGTAGATCGGA |
|  | antiBC2 | AGACAGATCGGA |
|  | antiBC3 | ACCAAGATCGGA |
|  | antiBC4 | AGTGAGATCGGA |
|  | antiBC5 | CATCAGATCGGA |
| Pri IC1-P7 | IC2P7 | CAAGCAGAAGACGGCATACGA |
| Pri IC1-P5 | IC1P5 | AATGATACGGCGACCACCGA |

Table S3: Kept loci and variant sites after filtering with populations in STACKS; presented for all calculated scenarios using the parameters p (minimum number of populations a locus must be present) and r (minimum percentage of individuals in a population required). The genotypes utilized in each dataset are also provided. The scenarios used for this study are highlighted in bold (referring to the datasets DS_INMAC_RAD01-04).

|  | Between species | | *A. declivis* | | *A. discapex* | | *A. laevapex* | |
| --- | --- | --- | --- | --- | --- | --- | --- | --- |
| Number of genotypes | 125 | | 48 | | 50 | | 23 | |
|  | Loci | Variant sites | Loci | Variant sites | Loci | Variant sites | Loci | Variant sites |
| p 1 r 0.1 | **23,856** | **12,254** | 7,884 | 5,495 | 13,143 | 5,185 | 6,451 | 2,365 |
| p 1 r 0.7 | 5,261 | 5,800 | 2,872 | 3,368 | NA | NA | 4,019 | 1,551 |
| p 2 r 0.1 | 3,556 | 7,836 | **5,064** | **5,148** | **4,829** | **4,617** | **4,283** | **2,207** |
| p 2 r 0.7 | 1,474 | 3,770 | 2,288 | 3,045 | 2,003 | 2,586 | 2,376 | 1,279 |
| p 3 r 0.1 | 1,224 | 3,559 | 4,092 | 4,809 | 3,636 | 4,146 | 3,204 | 1,748 |
| p 3 r 0.7 | 556 | 1,513 | 1,936 | 2,675 | 1,681 | 2,233 | 1,628 | 910 |

^
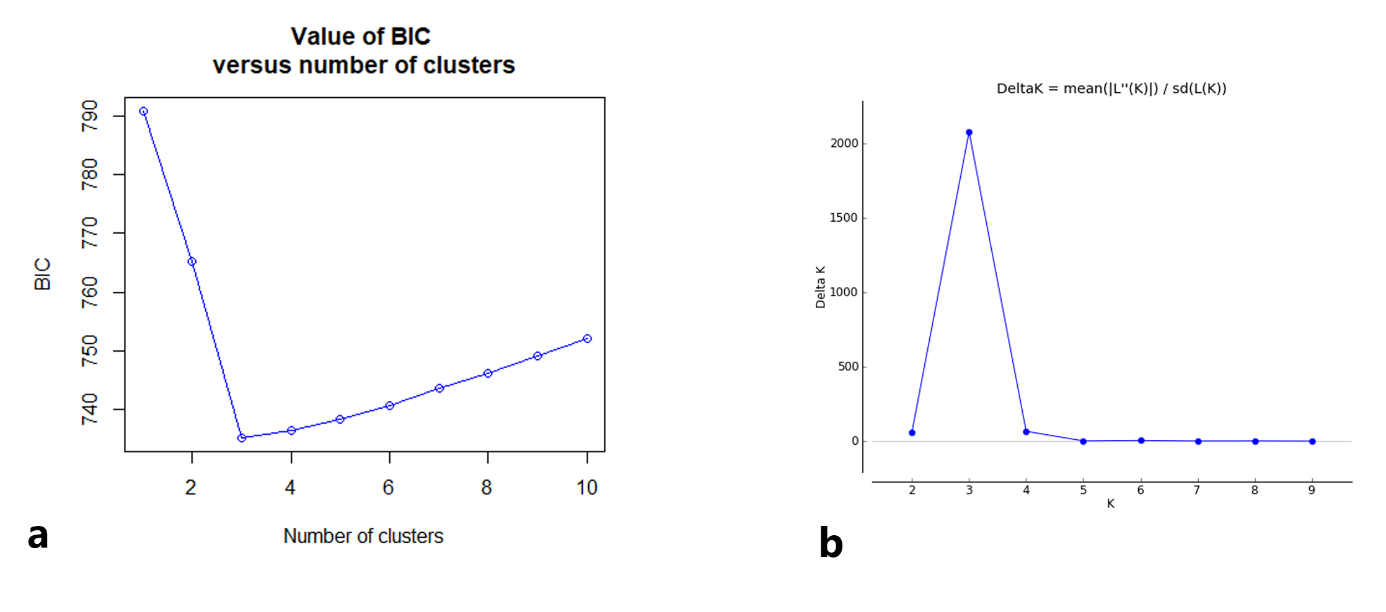
^

Figure S1: Result of the cluster analysis between species, searching for the most probable number of clusters K using 12,254 variant sites across 125 specimens of *Anatoma* by two different approaches: (a) DAPC (number of clusters K = 3) and (b) STRUCTURE Harvester (number of clusters K = 3).

^
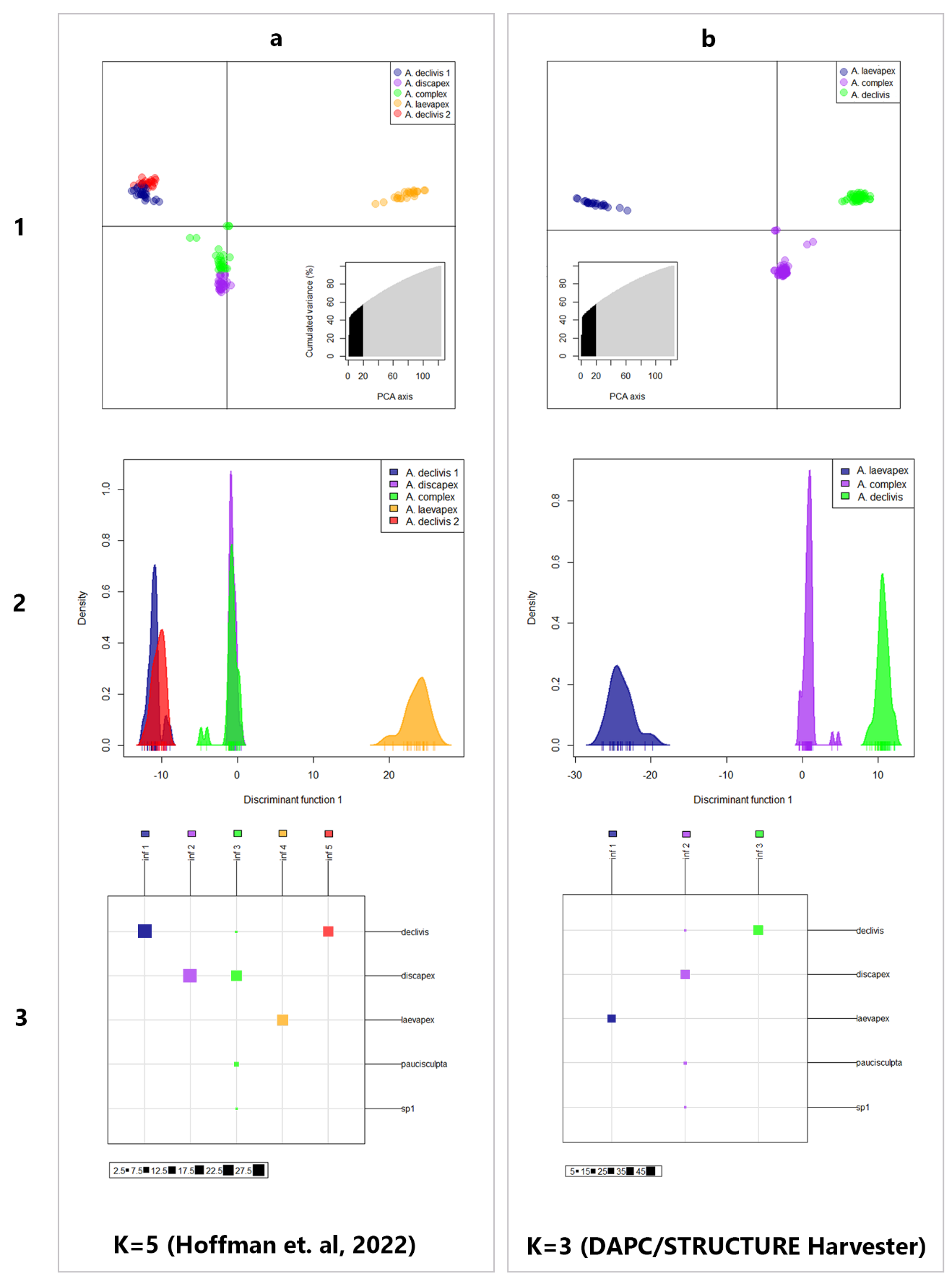
^

Figure S2: Result of Discriminant Analysis of Principal Components (DAPC). (1) Scatterplots drawn using 12,254 variant sites across 125 specimens of *Anatoma* in the R pack­age adegenet. Dots represent individuals, with colours denoting sampling origin. (2) Density plot of individuals along the first discriminant function from the DAPC are presented on the right. (3) Attribution of samples with their original identification to the inferred clusters (“inf”). Used clusters for the analysis: (a) a priori value of five expected clusters according to Hoffman et al, (2022), (b) de novo value calculated with DAPC and STRUCTURE Harvester of 3 clusters. Based on both approaches K = 3 and K = 5, *A. discapex* groups with *A. paucisculpta*, *Anatoma* sp. 1 and one specimen of *A. declivis*.

Table S4: Path sampling result for the three species delimitation models, considering only the three most abundant species and 3115 sites. All Bayes factor (BF) calculations are made against the current taxonomy model (runA). Therefore, positive BF values indicate support for model 1 (current taxonomy model). (MLE = Marginal likelihood estimate).

| **Model** | **Species** | **MLE** | **Rank** | **BF** |
| --- | --- | --- | --- | --- |
| RunA, current taxonomy | 3 | -73789.99921850911 | 1 | - |
| RunB, *A. declivis* and *A. laevapex* together (similar morphology) | 2 | -210790.54029908567 | 3 | 274001.1 |
| RunC, *A. declivis* and *A. discapex* together (based on similarities seen in COI) | 2 | -98847.3547792115 | 2 | 25057.4 |

Table S5: Path sampling result for the three species delimitation models, considering all five species and 61 sites. All Bayes factor (BF) calculations are made against the current taxonomy model (runA). Therefore, positive BF values indicate support for model 1 (current taxonomy model). (MLE = Marginal likelihood estimate).

| **Model** | **Species** | **MLE** | **Rank** | **BF** |
| --- | --- | --- | --- | --- |
| RunA, current taxonomy | 5 | -1404.3815246764614 | 1 | - |
| RunB, *A. declivis* and *A. laevapex* together (similar morphology) | 4 | -1409.4904014080316 | 2 | 10.2 |
| RunC, *A. declivis* and *A. discapex* together (based on similarities seen in COI) | 4 | -1632.7746545258497 | 3 | 456.8 |

Table S6: Abundances of the different species of the genus *Anatoma* used in this study (updated table of Hoffman et. al, 2022 according to results of this study).

| **Species** | **Vent Field 1** | **Gauss** | **Vent Field 2** | **Vent Field 3** | **Vent Field 4** | **Vent Field 5** | **Total** |
| --- | --- | --- | --- | --- | --- | --- | --- |
| *Anatoma* juvenile | 245 |  |  |  |  |  | **245** |
| *Anatoma* sp. 2 DZMB_2021_0096 |  |  |  | 1 |  |  | **1** |
| *Anatoma* sp. 1 DZMB_2021_0095 |  |  |  |  |  | 1 | **1** |
| *Anatoma paucisculpta* | 1 |  |  |  | 6 | 3 | **10** |
| *Anatoma laevapex* | 2 | 24 | 1 |  | 68 | 8 | **103** |
| *Anatoma declivis* | 18 | 5 | 11 | 7 | 148 | 16 | **205** |
| *Anatoma discapex* | 44 | 5 | 9 | 34 | 37 |  | **129** |
| unidentified | 4 | 1 |  | 1 |  | 1 | **7** |
| **Total** | **314** | **35** | **21** | **43** | **259** | **29** | **701** |


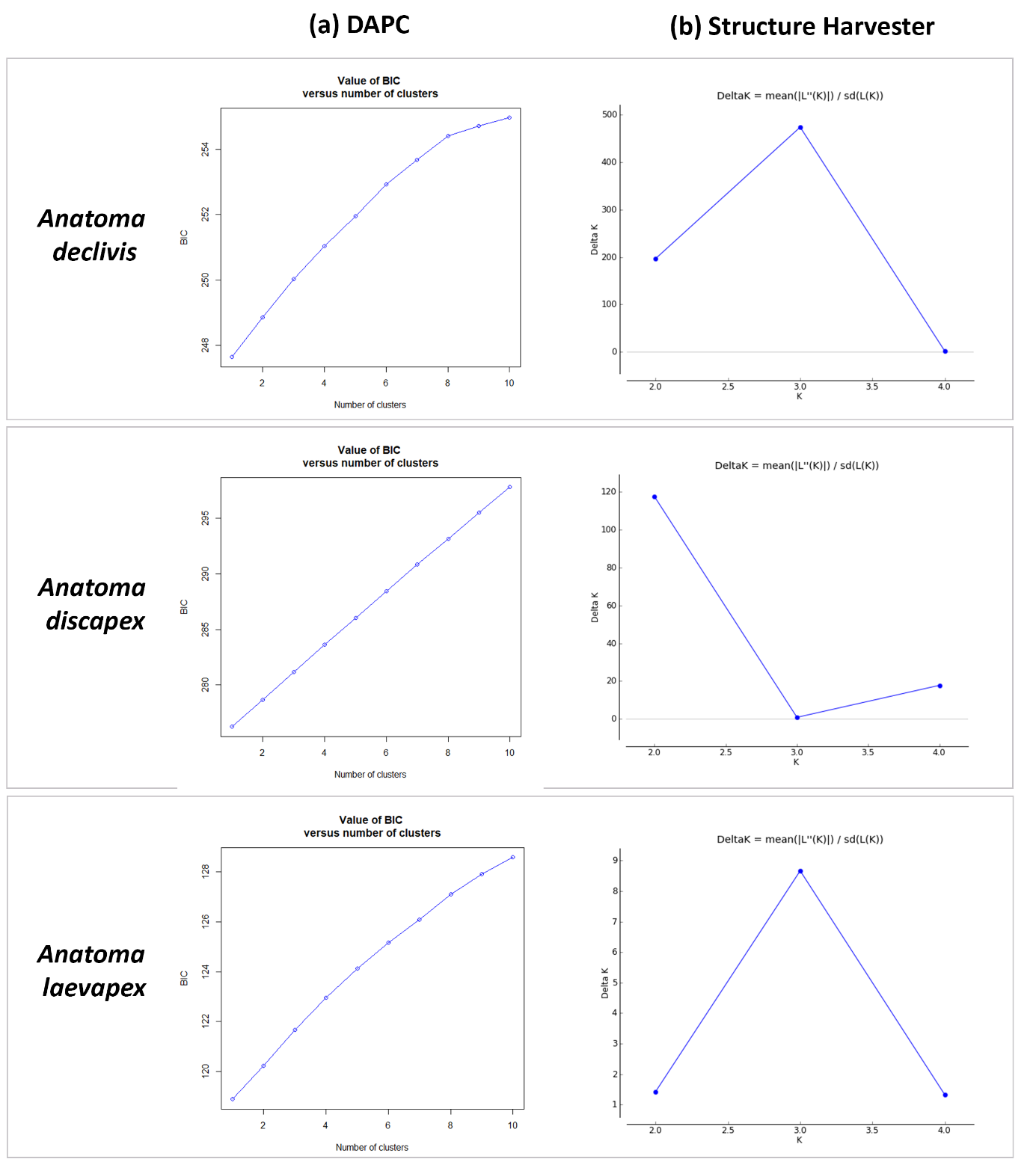


Figure S3: Result of the cluster analysis within species, searching for the most probable number of clusters K by two different approaches: (a) DAPC (number of clusters K = 1) and (b) STRUCTURE Harvester (number of clusters K = 3). The analysis was conducted on the three most abundant species *A. declivis* (using 5,148 variant sites across 48 specimens), *A. discapex* (using 4,617 variant sites across 50 specimens) and *A. laevapex* (using 2,207 variant sites across 23 specimens)*.*
